# Trusting our machines: validating machine learning models for single-molecule transport experiments†

**DOI:** 10.1039/d1cs00884f

**Published:** 2022-06-10

**Authors:** William Bro-Jørgensen, Joseph M. Hamill, Rasmus Bro, Gemma C. Solomon

**Affiliations:** Department of Chemistry and Nano-Science Center, University of Copenhagen Universitetsparken 5 DK-2100 Copenhagen Ø Denmark gsolomon@chem.ku.dk; Department of Food Science, University of Copenhagen Rolighedsvej 26 1958 Frederiksberg Denmark rb@food.ku.dk

## Abstract

In this tutorial review, we will describe crucial aspects related to the application of machine learning to help users avoid the most common pitfalls. The examples we present will be based on data from the field of molecular electronics, specifically single-molecule electron transport experiments, but the concepts and problems we explore will be sufficiently general for application in other fields with similar data. In the first part of the tutorial review, we will introduce the field of single-molecule transport, and provide an overview of the most common machine learning algorithms employed. In the second part of the tutorial review, we will show, through examples grounded in single-molecule transport, that the promises of machine learning can only be fulfilled by careful application. We will end the tutorial review with a discussion of where we, as a field, could go from here.

## Abstract 2

In this tutorial review, we will describe crucial aspects related to the application of machine learning to help users avoid the most common pitfalls.

## Graphical abstract

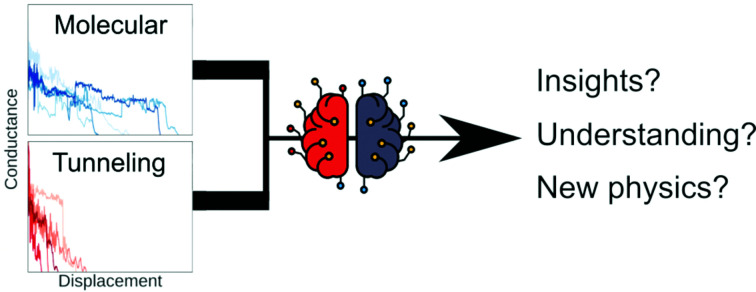

**Key learning points**

(1) Machine learning methods for single-molecule transport data analysis.

(2) Common challenges when using machine learning to analyse single-molecule transport data.

(3) Importance of sharing source code and experimental data.

(4) Perspective for future use of machine learning in single-molecule transport studies.

## 1 Introduction

Today, machine learning (ML) is effectively running victory laps in many areas of science, having given rise to new ways of analysing data and synthesising knowledge. While the underpinnings of ML can be traced back to 1800 with the development of the least-squares method and Bayes’ theorem, the advent of high-performance computers and large data sets, has decidedly provided a huge boost to the area in current years.

In particular, deep learning has provided a whole new way of solving problems and profoundly changed fields such as computer vision^1^ and natural language processing.^2^ The development of such advanced models heralds a new era of data analysis, but comes with the cost of increased obscurity as to what the models have learnt in order to function so effectively. Prediction alone does not grant us new insight.

Many fields, molecular electronics included, have seen an increased interest in using ML to analyse data. Molecular electronics, and specifically the field of single-molecule transport, spawned from an interest in using the unique material properties of organic molecules to solve challenges in conventional solid-state electronics. The two most common methods to measure single molecule conductance are the scanning tunneling microscope-based break-junction (STM-BJ) and mechanically controlled break-junction (MCBJ). Both methods form a nanogap by breaking a metallic point contact, but they differ in their approach to form the nanogap.

Briefly, the STM-BJ uses the existing functionality of a scanning microscope to form the nanogap in one of three ways: (1) the STM tip is held at a constant displacement from the substrate and waits for a metal-molecule-metal junction to form and break spontaneously; (2) the STM tip is brought into contact with an adlayer of the analyte and retracted; or (3) the STM tip is brought into contact with the substrate to form a metal-metal contact and then retracted. In the latter two cases a metal-molecule-metal junction is formed prior to the rupture of the junction and the process is repeated. The MCBJ forms and breaks Au-Au bonds in a thin metal lead mounted on an arching substrate.

While the field has come a long way towards making these measurements routine, the variability between measured samples tends to be high. Inherently, such variability is not problematic, but it necessitates statistics on a population level and makes it difficult to draw conclusions about the population from individual samples. The high variability also makes it difficult to discover and explore potential subpopulations in the data. Therefore, the hope is that ML will enable a more fine-grained analysis of the data from single-molecule transport experiments.

Despite the seemingly perfect match between the large data sets of single-molecule transport studies and the power of ML, some fundamental questions stand in our way. Due to the stochastic nature of the junction-forming process, we sample a large variety of different events. For example, different conformers of the molecule or samples where there is no measurable molecule between the electrodes. For a well-behaved molecule and with enough samples, we can estimate the average conductance of that molecule. Yet, on a per-sample basis, it is difficult to assign which event led to any particular sample.

From anecdotal experience, it is very common for a single experiment to contain a mixture of signals in the data that includes, at minimum, the following classes: traces in which no molecule was contacted and measured; unstable traces due to physical or electronic instabilities in the instrument; contaminant traces which contain molecule-like features; molecular traces from the analyte. It is also possible for the analyte to produce substructure alone, for instance, 4,4′-bipyridine.^3^

**Fig. 1:**
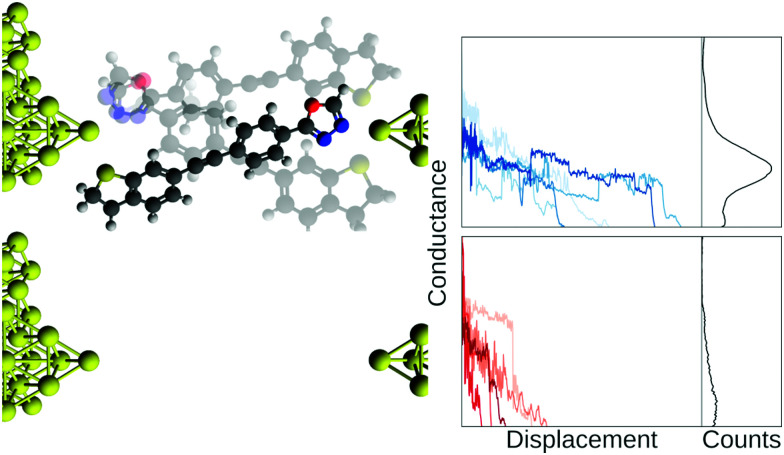
Illustration of a junction with and without a molecule bridging the electrodes. The blue traces are from a mechanically controllable break junction experiment with a molecule added and the red traces are from a similar blank experiment where no molecule has been added. In the right column, we show the full data set as 1D-histograms. Axes scales are arbitrary units. Conductance is normally on the order of nano Siemens and displacement is on the order of nanometers.

In Fig. 1, we show example traces from an experiment with a molecule (top row, blue traces) and without a molecule (bottom row, red traces). If we did not know which traces came from a blank experiment, it might be difficult to say whether or not a molecular signal is present in the red traces, although this is an area where future developments might provide a means of separating such traces. Compiling all our measured traces into 1D-histograms (right-most column, black) reveals that it is, indeed, only the blue traces that contain a molecular signal, seen as a peak in the 1D-histogram, but all information about each trace is now lost.

This challenge of assignment has led to a concern that if practitioners manually label samples, they might be at risk of confirmation bias. Such problems are compounded by the fact that it is relatively uncommon to make computational scripts and raw data openly available with publication. In part, this lack of sharing is due to a lack of metadata and formatting standards facilitating easy sharing of single-molecule transport data. Without open access to either the scripts or the data, analysis cannot be independently replicated and reproduced. Sharing, as we will argue, is also a way to minimise cognitive biases such as expectation or confirmation bias. As we will outline in this tutorial review, we have to be careful that ML helps us refine our definitions of molecular traces instead of cloud them.

We have structured the tutorial review according to the following: in the next subsection, we outline the history of single-molecule transport experiments to provide some intuition for the use of ML in molecular electronics. Then, we give a short introduction to common ML methods such as feature extraction, supervised learning, and clustering. The section after dives into the main problems associated with the use of ML. We start with a discussion of bias and the different ways it shows up in data analysis. Then, we move on to one of the most fundamental problems of ML: overfitting. After that, we will discuss how to build trust in the predictions of our ML model and how different choices of metrics might impact subsequent analysis. Then, we will talk about the use of unsupervised learning and its unique set of problems. After that, we outline how feature filtering can be used to optimise our models and gain a better understanding of what they learn. Finally, we will discuss the need for sharing of both source code and data. We end the tutorial review with a discussion of where we, as a community of aspiring ML scientists, might go from here.

### 1.1 Where is the field right now?

To understand where the field of single-molecule transport is today, it is instructive to look back at where the field started and how it has evolved. In the early days of the field of single-molecule transport, experimentalists were accustomed to designing, fabricating, and measuring “devices” that were hard to make, fragile, short-lived, and tested in highly controlled environments. In this paradigm, often only a handful of measurements could be performed on a single device, and comparisons between measurements were challenging. These handfuls of traces were also inspected individually. This paradigm informed the first attempts to measure single-molecule conductance.^4–6^

Inspired by studies of atomic contacts,^7^ assembling multiple traces into 1D-histograms lead to markedly improved signal-to-noise ratio by accentuating the molecular signal.^8,9^ Another important improvement was the use of 2D-histograms that retained displacement information.^10–12^

Yet, as we illustrated in Fig. 1, assembling traces into 1D-histograms comes at a cost. All time-dependent information is lost and any substructure in the data is disguised. Constructing 2D-histograms improves upon this, though information is still lost. For example, the contribution from each individual trace to characteristics in the 2D-histogram is difficult to characterize. Finally, it remained necessary, as with the individually inspected traces, to remove noisy and uncharacteristic/bad traces from the data set before analysis. For example, traces that only had a few data points or that only consisted of noise. Anecdotally, this filtering was often done by hand-selecting the ‘good’ traces. The histograms permitted more traces to be studied but were still limited by the number of traces an experimentalist could reasonably assess individually.

Consensus by 2005 was that one major challenge facing single-molecule electronics was experimental reproducibility.^13^ In subsequent years, incremental improvements of the measurement system, the data acquisition protocol, and the electronics were accomplished across the field in many laboratories. These improvements permitted laboratories to retain nearly all measured traces in analysis, removed the need to individually inspect all traces, allowed more traces to be retained, and increased the likelihood that more than a single characteristic signal was observed in the data analysis. The presence of different classes of trace in the data was better observed with richer representations of the data, the most common being 2D-histograms, that allowed for visualisation beyond simply individual traces and 1D-histograms. These new representations indeed revealed substructure in the data sets stemming from the presence of the four classes mentioned in the previous subsection.

Beginning with work by Lemmer *et al.*,^14^ interest increased in unsupervised methods in an attempt to tease out these substructures. Individually, each of the richer representations of the data mentioned above suggested the use of different unsupervised methods: the parameterisation of data led to vector-based reference parametrisation;^15,16^ correlation histograms led to the use of principal component analysis (PCA) to separate traces into different subgroups;^17–19^ finally, 2D histograms led to the use of image recognition methods.^20–22^

The end goals for employing advanced data analysis methods are based on two assumptions: (1) there are confounding signals in the data set that are uniquely different from the molecular signal, and therefore can be identified and removed in some objective manner; and (2) the molecular signal itself may contain multiple subclasses and these may also be grouped in some objective manner. Historically, these different signals were sorted by simple thresholding – either during pre- or postprocessing.

**Fig. 2:**
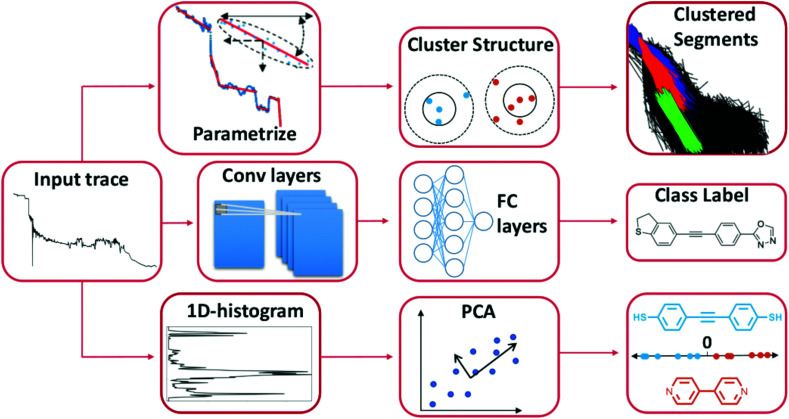
Three distinct ways to use ML to analyse single-molecule transport data. The first two examples uses unsupervised methods and the last one uses a supervised method. Top row: Each trace is parameterised by a series of linear segments that best describe each trace and subsequently a clustering algorithm is used to produce a hierarchical clustering structure where the linear segments that cluster together can be extracted.^23^ Middle row: By converting each trace to a 1D-histogram, principal components analysis (PCA) can be applied to the full data set. By then projecting each sample onto one of the first principal components, traces from two different molecules can be distinguished in an experimental mixture.^17^ Bottom row: The raw trace is pushed through a 1D-convolutional neural network trained on synthetic mixtures of two molecules. Traces from an experimental mixture of the same molecules can then be separated by the network.^22^

In Fig. 2, we illustrate some examples of how ML has been used to analyse data from single-molecule transport experiments. The input trace shown to the left is a typical measurement obtained from an MCBJ experiment. In the top row of Fig. 2, we provide a visual summary of what Bamberger *et al.*^23^ do. By approximating each trace with a set of lines, they can cluster these line segments using SOPTICS. This method allows them to extract similar regions from each trace. In the middle row, we illustrate the approach by Fu *et al.*^22^ Using a convolutional neural network trained on two experiments with two different molecules, they can separate samples from a single experiment with those two molecules. In the bottom row, we show the approach by Hamill *et al.*^17^ First, each trace is converted to a 1D-histogram and then PCA is performed on the full data set. This allows for a separation of the signals of two molecules from the same experiment by projecting onto one of the first principal components and assigning class labels based on whether the score is above or below zero.

The goals of molecular electronics parallel the goals of other fields, for example, analytical chemistry and computer vision. In common with these other fields, our ability to generate large amounts of data has surpassed our ability to adequately analyse the data. Furthermore, ML and the use of advanced data analysis in these fields have matured. These three examples demonstrate the diverse use and potential of ML in single-molecule transport. It is reasonable to assume that applying ML to data from single-molecule transport experiments will yield fruitful results in general.

We have done our best to provide a condensed overview of where single-molecule transport studies is coming from. Inevitably, with such a rich field, we have felt it necessary to exclude some references. For a more thorough introduction to the field of single-molecule transport studies, we recommend the excellent reviews that exist.^7,24,25^

## 2 Tools in the toolbox

This section introduces three overarching tools of ML: feature extraction, supervised learning and unsupervised learning. The purpose is to provide some common ground for all readers. We include feature extraction in this part of the tutorial review as it can make or break most ML algorithms.

Other areas that might be of interest to the community include self-supervised learning^26^ or semi-supervised learning.^27^ We will not discuss these hybrid techniques, but the reader should be aware that they combine aspects of feature extraction, supervised learning and unsupervised learning.

### 2.1 Feature extraction

Feature extraction is the process of creating derived features from raw input that are informative and reduce redundancy. The extent to which data needs to be represented in a new way depends on the field, data and objective.

For example, to predict the concentration of a UV/Vis-active molecule in an aqueous solution, the raw data is transformed from transmittance to absorbance. This transformation is a form of physics-guided feature extraction and is often enough to provide reasonable performance with linear models.

The same type of physics guided feature extraction is used in single-molecule transport experiments. Here, it is common to take the logarithm of the raw data and only look at conductance values between 1*G*_0_ and the noise floor of the instrument.

**Fig. 3:**
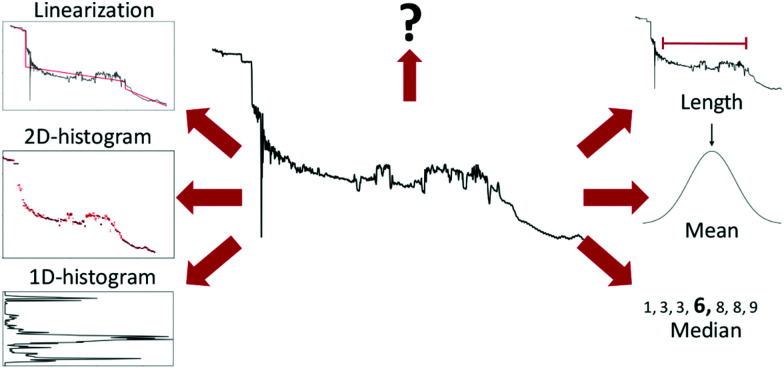
Examples of different ways to extract features from a conductance trace. Starting from the top right and going clockwise: Length of the molecular plateau; the mean of the conductance values; the median of the conductance values; 1D-histogram of conductance values; 2D-histogram of conductance values; approximating the conductance trace with several linear segments;^23^ a question mark to represent that, depending on the problem at hand, other features might have to be generated.

An example of such a lightly preprocessed sample is shown in the middle of Fig. 3. But, as shown by Fu *et al.*,^22^ such transformed data is often still too complex for linear models to achieve good performance. Thus, further feature extraction may be required for good performance.

Six different examples of ways to summarise a sample are shown around the trace in Fig. 3. The three methods (length, mean, and median) to the right summarise the trace with a single number, whereas the three methods to the left (1D-histogram, 2D-histogram and linearisation) are ensemble methods. The question mark symbolises that there are many more ways to extract features than depicted here.

Feature extraction can be valuable for several reasons: It can lower the computational cost of training; fewer parameters leads to a lower risk of overfitting; and it, potentially, makes it easier to understand what the model has learned. Feature extraction can also help remove irrelevant information. Even neural networks, which generally show good performance on raw data, benefit from a curated feature set.

Parallel, and arguably with some overlap, to the discussion of feature extraction is the discussion of data preparation. Decisions such as whether to normalize the data, how much of the noise floor to retain, or how to handle samples with a varying amount of data points all impact the performance of a model and any later analysis.

### 2.2 Supervised

Supervised learning is the task of training a computer algorithm to classify new, unknown samples based on training with a set of known samples.

Given a set of training data,{(**x**_1_,*y*_1_),(**x**_2_,*y*_2_),…,(**x**_*n*_,*y*_*n*_)} where each (**x**_*n*_,*y*_*n*_) is a pair from 𝒳 × 𝒴, the task of supervised learning is to learn a function *g* that maps from input space, 𝒳, to output space, 𝒴, *i.e.*, *g*: 𝒳 → 𝒴. Note that we will only be talking about supervised learning in a classification setting such that 𝒴 = {1,2,…, *K*} with *K* being the number of classes we wish to predict.

Many methods exist to generate labelled training data for a supervised classification algorithm. In computer vision, datasets are often annotated by people as humans are excellent at recognising objects in an image. While time-consuming, CAPTCHAs (Completely Automated Public Turing-test to tell Computers and Humans Apart) are an elegant solution to distribute the workload among a large number of people.

In single-molecule transport, we rarely have such a luxury. As explained in the introduction, a fundamental challenge is knowing what event a given experimental trace corresponds to. The community has come up with smart ways to generate training sets.^17,18,22^ For example, by performing separate experiments, though this is limited to distinguishing between individual molecules and cannot be used to extract subpopulations arising from the same molecule. It is also likely contaminated by tunnelling traces.

**Fig. 4:**
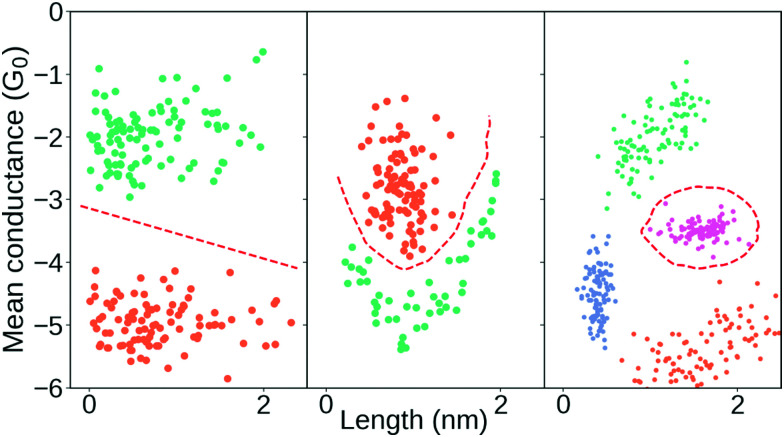
Illustration of three different decision boundaries in synthetic data. Points from the same class have the same colour and the red dashed lines correspond to hypothetical decision boundaries. Left: Linear decision boundary which, here in 2-D, consists of a line. Middle and right: Examples of non-linear decision boundaries.

One of the key defining features of a supervised learning algorithm is whether its decision boundary is linear or non-linear. In Fig. 4, we show three different decision boundaries on three different synthetic data sets. In the left plot, we have a linearly separable data set as we can draw a straight line that perfectly separates all the green samples from the orange samples. In 2D, this decision boundary is a straight line but generalises to a hyperplane in higher dimensions. In the middle and right plots, we show examples of two non-linear decision boundaries.

Clearly, models with a non-linear decision boundary can classify more complex data than models with a linear decision boundary, though the ability to model complex data is not free. Simple linear methods often allow for easy visualisation and interpretation of their estimated parameters, but such insight tends to be diminished as the complexity of the model increases. In many cases, even in situations where we primarily care about predictive performance, it is crucial that we can understand our model. For example, understanding allows us to draw conclusions about the data generating process and enables us to check that our model does not rely on spurious correlations. It also allows us to understand why a model might perform poorly and act accordingly. It even allows us to verify that the model is adequate for the specific sample at hand.

Often, it is difficult, or impossible, to gain insight into what a non-linear classifier has learned. It is a nontrivial task, in part because concepts such as ‘interpretable’, ‘explicable’ and ‘transparency’ are ill-defined. While the ML community is continually developing new methods to explain models,^28^ other researchers question the approaches taken and whether it is even necessary to explain our models. It is out of scope for this tutorial review to dive into the particularities of this discussion but we refer to the existing literature on the topic.^29–34^

#### 2.2.1 Linear

In the following, we make an explicit distinction between linear and non-linear classifiers. This distinction is here based on whether the decision boundary of a classifier decision is linear or non-linear. Though common, this distinction is somewhat arbitrary. While a linear classifier can be considered well-defined, a classifier can be non-linear in many ways; *e.g.* non-linear transformations of input variables, non-linear decision boundaries and variations thereof.

Linear classifiers are typically the simplest models and their decision boundaries are always linear (see left column of Fig. 4). In the left column of Table 1, we list a few of the most common linear classifiers. At the bottom, we have listed support vector machines (SVM) as both a linear and a non-linear classifier depending on the chosen kernel.

**Table 1:** A selection of common supervised algorithms

Linear	Non-linear
Linear discriminant analysis	Random forest
Partial least squares-DA	Gaussian process
Logistic regression	*k*-Nearest neighbor
SVM	SVM

Linear classifiers are often chosen as the baseline when comparing with other, more complex, methods. While non-linear methods often out-perform linear classifiers, it is not always the case if given structured data with meaningful features^35–37^ or if the samples do not adequately support a more complex model; *e.g.* if there are too few samples. While simple, many things – such as outliers and extreme inliers – are often easy to detect and diagnose for linear classifiers. There is also a notion of transparency as to what the model has learned.

With all classifiers, assumptions about the data are built into them. This is a prerequisite most commonly referred to as the “no free lunch”-theorem.^38^ Essentially, this theorem states that if no prior assumptions are made about the data we wish to predict, the average performance of our model will be equal to random guessing.

The assumptions can be rather general – such as the model being continuous, or similar samples originating from similar classes – or they can be strict. For example, linear discriminant analysis (LDA) behaves particularly well under the following conditions:

(1) Normally distributed samples. The class conditional variable, *x*, is sampled from a (multivariate) Gaussian distribution.

(2) Identical covariance matrices between each class, *i.e.*, the classes have to be homoscedastic; yet with different means.

If we only care about predictive performance, it is acceptable to apply a model on a data set where some, or all, of the assumptions are violated as long as we still obtain acceptable performance.^39^ If, on the other hand, we wish to infer something about the data generating process, we need to be more careful and use an appropriate model on a given data set.

#### 2.2.2 Non-linear

At times, a linear decision boundary does not cleanly separate the data. Then, it is reasonable to try a non-linear classifier if the data quality supports it as these models are more expressive in the boundaries they can model. Two examples of data that cannot be linearly separated are shown in the middle and right column of Fig. 4 along examples of non-linear decision boundaries. We list a few common non-linear classifiers in the right column of Table 1. Their greater ability to model complex interactions comes with a trade-off. For example, exactly due to their powerful modelling capabilities, they are prone to overfitting.

The use of more complex models can also come at the cost of transparency, though this is not always the case. For example, Gaussian process regression (or Kriging) is a commonly used non-linear model that originates from geostatistics. In Bayesian statistics, a Gaussian process prior can be used to build a regression model with continuous varying effects that can model data with non-linear effects. If an appropriate covariance matrix is chosen, it is possible to obtain a model that has good predictive performance. The estimated parameters can subsequently be used to infer characteristics about the data set and the population it has been drawn from.^40^

We also include neural networks (NNs) that are known as universal function approximators.^41^ While a necessary and crucial statement, it is of little practical use. If an NN has an infinite number of neurons, with an infinite number of connections between them, the network can approximate any function. In other words, if you can identify an unlimited number of lines in a picture, you can distinguish between all objects in that picture. We note that polynomial functions and the Fourier transform possess the same property of being universal function approximators.

NNs are an extremely powerful form of supervised learning, yet it is still an open question why they work so well.^42,43^ We have only listed a small selection of common NN-architectures in Table 2, but the literature is replete with different NN-architectures. NNs are the ultimate black-box ML algorithms; their predictive power is only rivaled by how difficult it is understand what they have learned.

**Table 2:** A selection of common neural network architectures

Neural network architectures
Feed-forward neural network
Convolutional neural network (CNN)
Recurrent neural network (RNN)
Autoencoder (AE)
Generative adversarial network (GAN)

### 2.3 Clustering

Clustering is concerned with grouping similar entities together. These methods tend to be used in two slightly different contexts:

(1) As part of an exploratory analysis where the goal is *e.g.* to discover potential patterns, spot anomalous data points or explore hypotheses about the data. This is an informal approach where we try to “let the data speak”.

(2) In a more rigorous setting where we already have an idea of the number of clusters yet, we have no labels on any samples.

In the single-molecule transport community, there is a hope that clustering can lead to a more unbiased analysis of our data.^16,20,21,23,44–48^ In Table 3, we list a few, common clustering techniques though many more exists.

**Table 3:** A selection of unsupervised algorithms

Clustering
*K*-Means + variations
OPTICS
DBSCAN
Finite mixture models

The practicality and usefulness of clustering is undeniable, but it has proven difficult to develop a precise, theoretical definition of clustering.^49^ The first challenge is how to define a cluster. Imagine the simplest definition of clustering with two requirements: dividing a data set into groups such that.

(1) Similar elements belong to the same group and;

(2) The members of each group are all similar.

While such a definition is intuitive, it is problematic.

Imagine a list of elements placed equally along a line. Upholding the first requirement that similar elements belong to the same cluster requires that every element goes into the same cluster. However, by doing that, we immediately violate the second requirement that every element of a group is similar.^50^ Such an impossibility theorem for clustering has been presented more formally by Kleinberg.^51^

In practice, unsupervised algorithms are still useful, but importantly it illustrates that these algorithms do not free us from making – potentially biased – assumptions about our data.

Most clustering algorithms favor internal homogeneity and external separation^52^ (also known as between-group and within-group variation, respectively). The fundamental question of “what defines a cluster? “ is a domain-specific requirement, not a statistical question. Hence, to perform meaningful clustering requires that domain insight guides the selection of appropriate clustering parameters.

This mainly means choosing what constitutes sample similarity. The model needs a measure of how similar two samples are before it can determine whether or not they belong in the same cluster. In certain situations, a physically motivated argument can be given for choosing a specific similarity score, but it is often difficult. Different definitions of similarity can have a considerable impact on the clustering result as we will show later. Simple testing of ‘all’ possible measures risks leading to selection bias, hence it is highly recommended to use domain knowledge for this.

Another challenge in clustering is that the user typically has to select the number of clusters. In most cases, this is a user choice rather than a fundamental property of the data; hence, the user has relatively free choice.

The single-molecule transport community has chosen several approaches: manual inspection looking *e.g.* for clustering with large inter-cluster distances or where each cluster has a significant difference in mean conductance,^20,21,23,45^ determining the number of clusters using statistical tools for internal validation^14,46,47^ or by using a clustering algorithm that automatically determines the numbers of clusters.^16,44^

Evaluating the quality of a given clustering structure against an external validation set is problematic. For one, we rarely have the ground truth labels. Even if we did, those labels imply that there is only one correct way to partition our data.

In contrast to external validation, internal validation uses only information already present in the data set. This is potentially also problematic as it does not evaluate the veracity of our clusters. Rather, the internal validation index compares which model (*i.e.* which clustering result) groups data points that are most similar, as defined by that particular internal validation index. For example, if we use an index that assumes a convex data set, it might be inappropriate if the data set is non-convex. A comprehensive analysis of internal validation indices has been performed by Arbelaitz *et al.*^53^

We note that the mere use of a clustering algorithm tends to assume the existence of more than one cluster. Why else would we reach for a clustering algorithm? Most algorithms can technically handle the possibility of a single cluster, but if the practitioner is hoping to find more than one cluster, the data might be represented in a way that emphasises more than one cluster – whether real or not.

## 3 A road lined with pitfalls

In this section, we dive into the use of machine learning in an applied setting and how to confidently draw conclusions from what our algorithms learn from our data. Our examples will mirror the use of ML in single-molecule transport analysis. At the crux of the matter is the issue of what event a trace corresponds to. While the uncertainty of that problem makes it difficult to apply ML techniques as they are normally applied, it does not make it impossible.

### 3.1 Defining bias

In the field of single-molecule transport, there is a concern that we may inject our own bias into the data analysis thus leading to subjective conclusions. Therefore, we should seek more unbiased methods, such as clustering algorithms.^15,16,21,44–46,54^

The term bias means a systematic deviation from the true value and covers a multitude of problems ranging from statistically derived biases to biases coming, *e.g.*, from how the samples were gathered or analysed. We will describe the more common types of biases and their importance.

In statistics, bias is a well-defined concept. If we have an unbiased estimator, the expectation value of that estimator will be equal to the true value of our estimated parameter. For instance, the sample mean, 
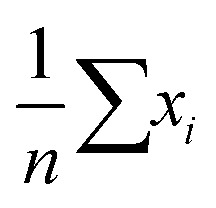
, is an unbiased estimator of the population mean, *μ*, for a series of independent and identically distributed observations drawn from a normal distribution.

While an unbiased estimator is desirable, it is not required to get a good estimate of our parameter of interest. For example, a potentially biased estimator of the population mean is given in eqn (1):1
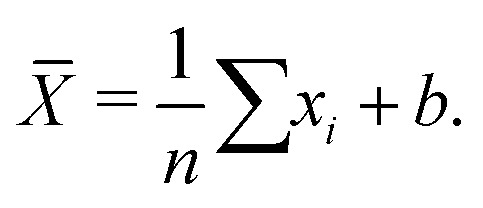
Here, *X̄* is a biased estimate of the population mean, *μ*, with *n* samples, *x*_*i*_ a measurement value and *b* is the bias term. If 
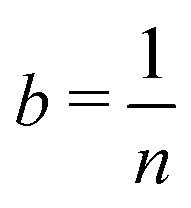
 then as *n* → ∞, *X̄* will approach *μ*, the true value of the population mean. It is not unbiased, but it is consistent – that is, as the number of data points increases, our estimated parameter will converge on the true value of the parameter. We will refer to such bias – exemplified here with the parameter *b* – as statistical bias.

In classical statistics, the main concern was having unbiased estimators. The total error of an estimate consists of the systematic part – bias – and the random part – variance. In many modern settings, the error coming from variance can easily be orders of magnitude larger than the bias term and there are therefore many tools that actively bias the estimate a little, in order to gain a huge reduction in variance and hence a much lower total error.

In ML, it is common to bias our estimator, or model, through the use of regularisation – known as shrinkage in statistics. While such regularisation will increase the error from the bias term, it will make our model less sensitive to the random noise of the data thus improving the performance of the model. Regularisation is a balance as it might also mean that the model loses its ability to accurately model the data. This balance is known as the bias-variance trade-off.

We refer to the next type of bias as measurement bias. A few are listed in the left column of Table 4. These are all related to the collection, handling and analysis of data. Imagine that we wish to estimate *μ* of a normal distribution using eqn (1). If our instrument is not sensitive enough to measure samples from part of the distribution, our estimate of *μ* will be biased. Such a detection bias will persist even if we collect more samples. These types of biases are often avoided by changing the experimental setup or changing the framework in which we analyse our data. Other times, they are unavoidable and we instead have to assess how they might impact our analysis.

**Table 4:** A selection of measurement and cognitive biases

Measurement	Cognitive
Detection bias	Confirmation bias
Selection bias	Base-rate neglect
Survivorship bias	Hot-stuff bias

Finally, psychology has unearthed a litany of cognitive biases. Generally, they help us make sense of the world at a reasonable pace, but they can also cause us to make erroneous conclusions. In the right column of Table 4, we have listed a few cognitive biases. These are separate from the other two types of biases though they share some traits. For instance, few cognitive biases are mitigated by having more data. In fact, the reiteration effect suggests that repeated exposure might make us more susceptible to believe false information.

Analogous to the discussion of cognitive biases, and arguably with some overlap, is that of logical fallacies. These errors in reasoning can bias us towards conclusions our data cannot substantiate. While out of scope for this tutorial review, they are important and examples include ‘affirming the consequent’ and ‘appeal to probability’.

In the preceding, we have outlined different contexts where the concept of ‘bias’ appears. In the following sections, we use the described types of biases to explain the sources of errors that are common in ML.

### 3.2 (Over)fitting supervised models

Overfitting is one of the most fundamental problems of ML and it is both ubiquitous and subtle. It is intuitively easy to understand yet there is no single solution for this problem. Overfitting does not even have to occur during the training of the model as it can happen during model selection.^55^ The overfitting problem leads to models that seem satisfactory when tested in a controlled setting, but when put to the test on unseen data – for example, from a new experiment entirely – their performance drops significantly.

The opposite problem also exists where a model is not powerful enough to model the relevant variation in the data. This problem, called underfitting, also results in performance degradation of our models. With the recent development and widespread use of powerful ML techniques, underfitting is, in practice, less of a problem than overfitting. We will provide examples of both regimes. In the following, we will work in a regression setting due to ease of illustration. All conclusions also hold for classification.

**Fig. 5:**
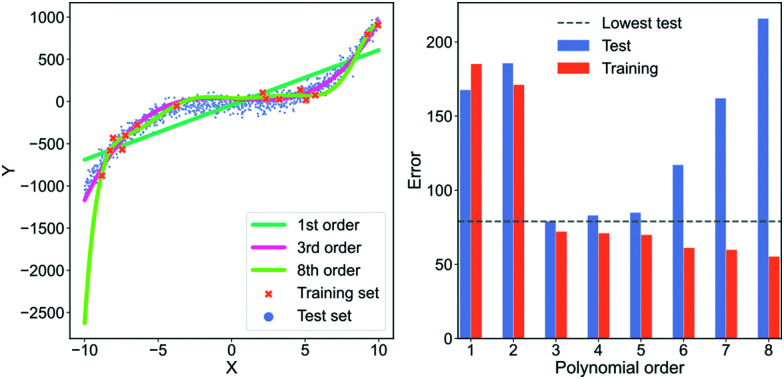
An example of over- and underfitting by fitting increasingly higher-order polynomials to simple, synthetic data. The data has been generated from a 3rd order polynomial with Gaussian noise subsequently added. Left column: Predictions from a 1st (green line), 3rd (orange line) and 8th order polynomial (dark blue line) *vs.* the true value. Red crosses are samples that each model was trained on, blue dots are the samples that the model is tested on. Right column: Error on the training set (blue) and error on the test set (red) for fits of increasingly higher-order polynomials. The horisontal dashed grey line indicates the lowest test set error.

In Fig. 5, we show an example of fitting higher and higher order polynomials to a synthetic data set. The data has been generated according to

2 *y*_*i*_ = *x*_*i*_^3^ + 

<svg xmlns="http://www.w3.org/2000/svg" version="1.0" width="23.000000pt" height="16.000000pt" viewBox="0 0 23.000000 16.000000" preserveAspectRatio="xMidYMid meet"><metadata>
Created by potrace 1.16, written by Peter Selinger 2001-2019
</metadata><g transform="translate(1.000000,15.000000) scale(0.014583,-0.014583)" fill="currentColor" stroke="none"><path d="M880 920 l0 -40 -40 0 -40 0 0 -80 0 -80 -40 0 -40 0 0 -40 0 -40 -40 0 -40 0 0 -80 0 -80 -40 0 -40 0 0 -80 0 -80 -40 0 -40 0 0 -80 0 -80 -80 0 -80 0 0 -40 0 -40 -80 0 -80 0 0 80 0 80 80 0 80 0 0 40 0 40 -80 0 -80 0 0 -40 0 -40 -40 0 -40 0 0 -80 0 -80 40 0 40 0 0 -40 0 -40 80 0 80 0 0 40 0 40 80 0 80 0 0 40 0 40 40 0 40 0 0 80 0 80 40 0 40 0 0 80 0 80 40 0 40 0 0 80 0 80 40 0 40 0 0 40 0 40 40 0 40 0 0 -120 0 -120 -40 0 -40 0 0 -200 0 -200 40 0 40 0 0 -40 0 -40 40 0 40 0 0 80 0 80 40 0 40 0 0 80 0 80 40 0 40 0 0 160 0 160 40 0 40 0 0 40 0 40 40 0 40 0 0 40 0 40 -40 0 -40 0 0 -40 0 -40 -40 0 -40 0 0 -40 0 -40 -40 0 -40 0 0 -160 0 -160 -40 0 -40 0 0 320 0 320 -40 0 -40 0 0 -40z"/></g></svg>

(0,1) × *ε*

where *x*_*i*_ is drawn from a uniform distribution with *x*_*i*_ ∼ 𝒰[−10,10) and *ε* is a parameter for noise. We set *ε* = 3. We generate a training set with 15 samples (red crosses in Fig. 5) and a test set with 1000 samples (blue dots in Fig. 5). Using the training set, we fit eight polynomials of higher and higher order and evaluate their performance on the test data. To visually inspect what the models fit, we plot the lowest order model (green line), the 3rd order model (orange line) and the highest order model (dark blue line) in the left plot of Fig. 5. To quantify the performance of each model, we plot the root mean squared error of both the training and test set in the right plot of Fig. 5. In the plot on the right, the red bars are the error on the training set, the blue bars are the error on the test set and the dashed, grey line is the lowest error on the test set. In the ESI,† we show the average training and test error for 1024 experiments in Section “Training and test errors on average” of the ESI.†

In the left plot of Fig. 5, we see that the highest order polynomial describes the training set reasonably well yet fails to capture the significant trend in the test set. This discrepancy in performance is also highlighted in the plot on the right of Fig. 5. Here, the test error for the 8th-order polynomial is the second-highest despite it having the lowest training error. This is a typical case of overfitting. The model has fitted the noise in the training data instead of the overall trend.

If we look at the 3rd-order polynomial it does well at capturing the significant variation of the data without modelling the noise. This is also reflected in the right plot of Fig. 5 where the 3rd-order polynomial has the lowest test error but has neither the highest nor the lowest training error. Finally, we see that the lowest order polynomial is a straight line that only captures the overall sloped trend in both the training and test set. It does not capture the slight curvature present in the data which results in an underfitted model. From the plot to the right in Fig. 5, we see that the underfitted model results in poor performance on both the training and test set.

This example highlights that the training error for large, complex models is a poor indicator of real-world performance. In most cases, the error on the training set should primarily be used as a diagnostic to ensure that our model is not collapsing or diverging. The more free parameters a model has, the more pronounced the overfit can be. Deep neural networks are extreme in terms of their amount of free parameters, making them susceptible to overfitting. However, simpler models can also overfit, as we have seen in this example.

The models shown in Fig. 5 are also examples of models with differing degrees of variance and bias. The 1st-order polynomial is almost agnostic to the training data and only models part of the systematic variation in the data. This model has a high bias and low variance. On the contrary, the 8th-order polynomial has a high variance and low bias and is thus highly sensitive to any idiosyncrasies of the data because the excess degrees of freedom are used on modelling the noise in the data. As mentioned in Section 3.1 “Defining bias”, an effective way to avoid overfitting, *i.e.*, reduce the error from the variance term, is by regularisation.

While the example of Fig. 5 illustrates the effect of under- and, in particular, overfitting, it is instructive to look at different causes of overfitting and to understand how to combat each type.

#### 3.2.1 Misunderstanding of the principle of holdout sets

A holdout set is a subset of the data that has no overlap with either the training set or the test set. It is set aside as early as possible, preferably before looking at the data. A more laborious alternative is to perform a new experiment and save those measurements as the holdout set.

Assuming the training data is truly representative, it is a common and sound principle to validate our model using a holdout set. However, imagine that you have performed variable and feature selection, tuned modelling parameters, considered data transformations, *etc.* If the holdout set is created after such choices have been made, it is indeed no longer a holdout set. You may have artificially created a situation where your data set has now been transformed into a simpler, better-behaving problem where seemingly good results bear no meaning for real world use. For this reason, it is important to set aside the holdout set as early as possible.

#### 3.2.2 The cross-validation trap

Cross-validation (CV) is a data-efficient way to simulate a holdout set. One variant of CV is to partition our data set into *k* non-overlapping subsets. The model is then trained on each combination of *k* − 1 subsets and tested on the last subset.

It is beneficial as all samples get to be part of the training data and also get to be part of the simulated hold-out data set. Hence, CV may provide more precise estimates of the performance than a holdout set if the holdout set is small.

However, proper CV depends heavily on the purpose of the model. If you have data with a temporal component – for example, if you wish to predict one segment of an MCBJ trace from another segment – you need to be careful. A naïve approach to CV would pay no attention to the chronological ordering of each segment. It is then possible that the model is asked to predict a segment from the past using a segment from the future. Given that the model does not have access to future data when deployed, it is a nonsensical task to validate against and might obscure the real performance of the model.

Properly implemented CV can lower the risk of an overfitted model but it requires a thoughtful implementation. Inappropriate use of CV can lead to overfitting. CV is related to the task of finding the best model, or hypothesis, out of many and it can be shown that this task can lead to picking an overfitted model.^56^

#### 3.2.3 Overly simple training data

Imagine a data set that is not representative of the true complexity of the use cases. Perhaps the training data is based on simulated data that only approximate data from the real world, or the data comes from a single lab even though the model is intended to be deployed in many labs. In such cases, you may be able to fit the specifics of the training data well, but the predictive power will be limited.

Unfortunately, neither holdout sets nor CV can help diagnose overly simple training data. The best way to counter this type of overfitting is typically a combination of detailed domain knowledge and effort to obtain more challenging test data that better represent the real complexity of the use cases.

In the ESI,† we list a few more considerations that a practitioner has to think about to avoid the risk of overfitting.

In the preceding, we have talked about overfitting in the context of classical ML. These challenges also hold for deep learning though there are indications that more complex regimes might exist. For instance, in specific situations, more data might hurt model performance.^57^

### 3.3 Building trust in our models

If we wish to use our model to make predictions about unseen data, we need to be confident in its performance and adequacy for the type of data it will be used on. To build that confidence, we need to choose some metrics to evaluate our model against. Often, single-molecule transport studies, people choose single-threshold metrics, such as accuracy, to evaluate their supervised model.^18,22,45,48,58^

Choosing accuracy can be beneficial as it is intuitive, but it assumes the cost of misclassifying false positives and false negatives are equal. This assumption is not always sufficient as we will illustrate in the following example.

Imagine a food company that produce chocolate bars. The company is situated in a country with strict food laws so for any bad chocolate bar in violation of these laws, the company is fined €1000. Discarding a good chocolate bar only costs €1 in lost profits.

The company wants us to develop an ML model that, given some sensory data, can predict whether or not any given chocolate bar has to be discarded. Clearly, misclassifying a bad chocolate bar as being good is many times more costly than misclassifying a good chocolate bar as being bad. In other words, the cost of a false positive (bad chocolate bar being good) is much higher than a false negative (good chocolate bar being bad).

If we validate our ML model using accuracy, we implicitly evaluate our model under the assumption that the cost of false positives and false negatives are equal. This assumption does not hold for the quality assurance of the chocolate bars and, as we will argue, might not be the case either for discerning between molecular and tunnelling traces.

The cost of misclassification is always context-dependent. In the case of chocolate bars, we wish to reduce the expenses of complying with food laws. In the case of classifying traces for later analysis, we posit that it is worse to classify tunnelling traces as molecular traces rather than the opposite. This is an explicit choice that can be justified based on prior knowledge about the data but is not statistically based. While such a choice might seem subjective and arbitrary, it is also essential and inescapable. It is not avoided by using accuracy, rather it makes it implicit. The balance of false positives and false negatives depend on the properties we investigate and the conclusions we wish to draw.

**Fig. 6:**
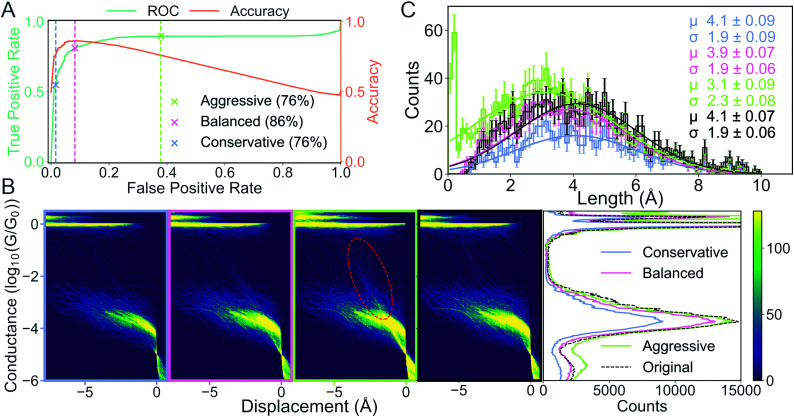
How choice of metrics impact subsequent analysis: the case of separating molecular and tunnelling traces. (A) False positive rate (FPR) *vs.* true positive rate (TPR) (green line) and FPR *vs.* accuracy (orange line). The three crosses and their corresponding dashed lines (grey, pink and light green) represent three different decision threshold levels: conservative, balanced and aggressive. The percentages in the legend lists the accuracy at each threshold. (B) Histograms of traces labelled “molecular”. Four 2D conductance *vs.* electrode separation histograms (from left to right) for the conservative, balanced, aggressive decision threshold and the true distribution, respectively. The red, dashed ellipsis highlights that a significant number of tunnelling traces have been misclassified as molecular. Final plot shows 1D conductance histograms at each decision threshold and for the true distribution. (C) Distribution of lengths of the molecular traces at each decision threshold at their respective colours and for the true distribution in black. The solid lines are fitted Gaussians with mean (*μ*), standard deviation (*σ*) and standard error for each given in the legend text.

In Fig. 6, we show how the results of subsequent analysis depends on the initial choice of metrics that were used to validate the ML model. The data set has kindly been provided by Magyarkuti *et al.*^18^ Using an MCBJ setup, they measured traces of 4,4′-bipyridine at 4.2 K, a molecule that has previously been shown to exhibit a double-step molecular plateau at room temperature.^10^ To be able to quantify the performance of their unsupervised method, they hand labelled all traces depending on whether there was a molecular signal (1863 traces) or not (3219 traces). A few examples from each class are shown in the ESI.† To avoid biasing the classifier towards the majority class (tunnelling traces), we have balanced the data set by removing 1356 tunnelling traces. We refer to the hand labelled labels as the “true” labels and to the balanced data set as 4K-BPY. We use linear discriminant analysis (LDA) as our model with default settings as implemented in scikit-learn. There is no optimisation of hyperparameters.

As input to our model, we transform the traces as follows: first, we discard any data point above −0.5*G*_0_ and below −5.5*G*_0_. Second, we truncate our traces to only include the first 750 data points. Lastly, we convert each trace into a 2D-histogram of 32 × 32 bins.

We have deliberately chosen a subpar model and feature set to illustrate the impact of different decision thresholds. In the ESI,† we show an example of a model that performs almost perfectly making our choice between different decision thresholds less significant.

We measure the performance of our model with two metrics: accuracy (orange curve in Fig. 6A) and the receiver operating characteristic (ROC) curve (green curve in Fig. 6A). The ROC curve assesses false positive rate (FPR) *vs.* true positive rate (TPR) at every decision threshold of the model. TPR is also known as sensitivity or recall while FPR is defined as 1 – specificity. A model with a ROC curve close to the diagonal is no better than random prediction. Conversely, the closer the ROC curve is to the upper left corner, the better the model performs. A summary of the ROC curve can be given by the area under the ROC (AUROC) and tells us, averaged over all decision thresholds, what is the performance of the model. The AUROC is equal to the probability that a classifier will rank a randomly chosen positive instance higher than a randomly chosen negative example. Alongside the ROC curve, we also plot the accuracy calculated at the same decision thresholds as the ROC curve.

To illustrate the challenges with single-threshold metrics, we choose three different thresholds: Conservative, balanced and aggressive (blue, pink and light green crosses in Fig. 6A, respectively). If we maximise accuracy, *i.e.*, balance TPR and FPR equally, we get the balanced threshold.

The aggressive and conservative thresholds both have an accuracy of 76%, but their FPR differ significantly. The conservative threshold has an FPR of 2% whereas the aggressive threshold has an FPR around 38%. Conversely, the conservative threshold has a TPR of about 55% whereas the aggressive threshold has a TPR of almost 90%. This difference in TPR means that the conservative threshold will classify very few, if any, tunnelling traces as molecular traces, but classify a significant portion of molecular traces as tunnelling traces. The aggressive threshold will correctly classify almost all the true molecular traces at the cost of falsely classifying some true tunnelling traces as molecular traces. The balanced threshold has an FPR of 8%, a TPR of 81% and an accuracy of 86%.

In Fig. 6B, we plot, as 1D- and 2D-histograms, the traces that have been classified as molecular for each of the three thresholds and the true labels. In the three columns to the left, we have plotted 2D conductance *vs.* electrode separation histograms for the conservative, optimal and aggressive threshold, respectively. In the fourth column, we have plotted molecular traces according to the true labels. The traces have been aligned at the crossing of 5 × 10^−5^*G*_0_. In the fifth column, we have plotted the 1D-histogram for each threshold and the true labels.

The biggest difference in the 2D-histograms is the appearance of diffuse, diagonal lines that are most clearly seen in the red encircled region. These diagonal lines are indicative of tunnelling traces being labelled as molecular traces, *i.e.*, there is a significant number of false positives.

In the 1D-histograms, we see that the average conductance depends very little on the decision threshold. We also see that the main peak at −3.8*G*_0_ is lowered slightly when choosing the conservative threshold instead of the aggressive one. This is expected as the conservative threshold will classify fewer traces as molecular.

In Fig. 6C, we plot the length distribution for the traces classified as molecular at each threshold. We fit a Gaussian function to each distribution and, in the upper right corner of the plot, we have provided the mean (*μ*), standard deviation (*σ*) and their uncertainties for each fit. The error of each bin is assumed to be Poisson distributed. We can see that the average length for the junctions changes from 3.1 ± 2.3 Å for the aggressive threshold compared with 4.1 ± 1.9 Å for the conservative threshold. This change in mean length is because the conservative threshold has misclassified fewer tunnelling traces as molecular traces.

In this example, we explicitly deal with the problem of an imbalanced data set by removing ∼48% of the tunnelling traces. Such an imbalance might introduce a significant bias towards the majority class. Imagine a classifier trained on a data set with 99% negatives and 1% positives. It is trivial to obtain 99% accuracy if the classifier only predicts the negative class.

Neither ROC curves nor the accuracy score is equipped to deal with an imbalanced data set, but several other methods exist. We can up-sample the minority class, down-sample the majority class, or use performance metrics that explicitly encode a notion of imbalance such as Cohen's kappa or a precision-recall curve. Each approach has distinct strengths and weaknesses. For example, by down-sampling the majority class, we obtain a more balanced data set at the cost of discarding examples of the majority class. Nonetheless, what we have shown in this section holds generally and does not directly depend on whether the data set is balanced or not. One issue with ROC curves is that they can only be used for binary classification. There are extensions beyond two classes though this extension is nontrivial.

To trust our models, we need to evaluate them by consciously chosen metrics. If we assume an equal cost between false negatives and false positives, accuracy is the right choice. Yet, as we have illustrated in this example, it is not universally the right choice. For a binary classification task, plotting the ROC curve and reporting the AUROC is often a better choice as it provides information about the TPR and FPR across all decision thresholds. It also forces us to make an explicit decision about the FPR and TPR that we tolerate in our model. While we have only looked at the ROC curve and accuracy, a systematic comparison of 17 performance metrics has been performed by Ballabio *et al.*^59^

### 3.4 Predicting is not explaining

In single-molecule transport studies, it is often difficult to classify individual traces according to whether they belong in the molecular or the tunnelling class. Even more so, we might want to classify traces that belong to different conformers of the same molecule. While the problem of manually labelling a few thousand traces is partly motivational, it also raises the concern that we, as humans, might not objectively label the traces. To solve this problem, the community has set their eyes on clustering techniques and unsupervised ML.

Clustering is unbiased in a statistical sense and it is not possible to overfit models in the same way as with supervised methods. On the other hand, it has no mechanism to prevent or detect measurement biases and the user of a clustering algorithm can still fall prey to cognitive biases and logical fallacies. As we will show in the following, clustering algorithms require careful use to avoid spurious claims. A more general analysis of some of the challenges of using clustering on high-dimensional, biological data has been performed by Ronan *et al.*^60^

In the following, we have chosen a single clustering algorithm with different similarity measures to show that our results are heavily dependent on our choice of similarity metric.

**Fig. 7:**
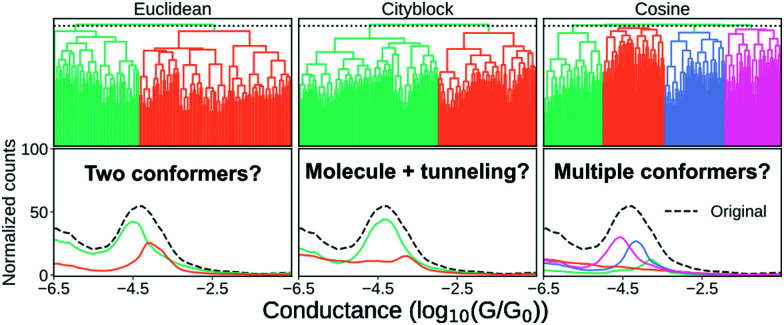
Clustering results for a one-molecule data set using complete-linkage and three different distance metrics: Euclidean, city block and cosine. The top row shows the hierarchical clustering as a dendrogram and the bottom row shows the 1D-histograms for each cluster (coloured lines) and the original dataset (black, dashed line). For visual clarity, we omit some of the lower nodes to condense the dendrogram. This omission has no impact on the clustering result.

In Fig. 7, we show three different clustering results on the data set introduced in the ESI,† from an MCBJ experiment with one type of molecule in solution. We use agglomerative clustering with complete linkage for all three results, but we change the distance metric used to measure the pairwise similarity between samples. The first column of Fig. 7 uses Euclidean distance, the second column uses city block distance and the third column uses cosine distance. As input, we convert every sample into a 1D-histogram with 128 bins and conductance values from −6*G*_0_ to −0.5*G*_0_.

In the top row, we plot the dendrogram for each clustering result. A dendrogram visually represents the similarity between two samples as the height of the lowest internal node they share. For visual clarity, we omit some of the lower nodes to condense the dendrogram. This omission has no impact on the clustering result.

In the bottom row, we plot the 1D-histogram of the obtained classes by cutting the dendrogram at the dotted black line. In the ESI,† we plot the results for two more feature vectors: One where each trace is converted into a 2D-histogram of 32 × 32 bins and another which appends the 1D-histogram to the 2D-histogram feature vector.

We see in Fig. 7 that our clustering result depends considerably on our definition of similarity. With Euclidean distance, it looks like the molecule has a high and a low conducting state whereas using city block distance suggests only one dominant binding configuration alongside some tunnelling traces. Cosine distance suggests multiple clusters which could be explained by multiple conformers. All three clustering results are intrinsically valid yet highly dissimilar.

To move forward, we need to pick one of the solutions as the correct one. One method the community has chosen is, essentially, visual inspection.^20,44^ Analogous to what we have done here, the clustering result is validated by outlining a plausible situation that could have given rise to the subpopulations. From what we have shown in the preceding, it should be clear that this cannot be a sufficient explanation for why a clustering result is valid. Validation has to be more than just explaining the clusters.

Another approach is to first show that the clustering algorithm works on synthetic data and then use the algorithm on real data.^21,45–47^ While alluring, this approach is also problematic. As we mentioned in Section 3.2 “Overfitting supervised models”, synthetic data is often simpler than real data. Analogous to a complex model performing well on simple data, a clustering model performing well on synthetic data does not mean that it applies to real data. Unless using highly elaborate synthetic data, validation on synthetic data can tell us that the algorithm works, but not much more than that. Validation on real data is essential.

**Fig. 8:**
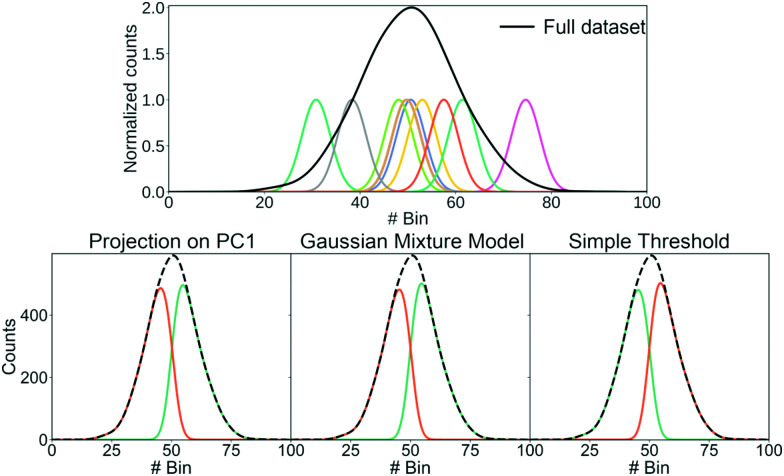
Illustration of how different clustering techniques split normally distributed data. The top row shows each sample (green) and the full dataset (black). The bottom row shows the resulting clusters (green and orange) using three different clustering techniques: Projection of each sample onto the first principal component and determining whether the score of each sample is higher/lower than 0; using a Gaussian mixture model with two components; using a simple threshold.

Having shown that clustering does not necessarily give unique results, we will try to explain part of the problem. In Fig. 8, we have generated a 1D histogram (black line) from 2000 individual Gaussian peaks, each representing a histogram of individual traces. A handful of individual samples (coloured lines) is also shown. Each sample is modeled as a Gaussian:3
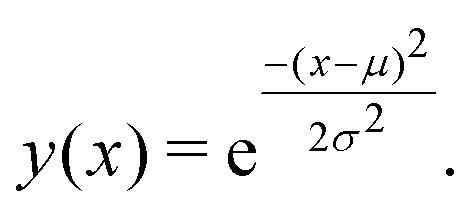


Here, *μ* is the mean of the Gaussian and *σ* is the standard deviation. To generate the 2000 samples, we let *x* = {0,0.1,0.2,…,100}, *σ* = 3 and then we sample *μ* according to a Gaussian distribution:

4 *μ* ∼ 𝒩(50,10).

Consequently, we have 2000 samples with a Gaussian-shaped signal (eqn (3)) where *μ* for each of these signals are drawn from a Gaussian distribution (eqn (4)).

We use three different methods to cluster the 2000 samples into two classes. The first method uses PCA where each sample is projected onto the first principal component. A score above 0 puts the sample into the first class and a score below 0 puts it in the second class; the second method uses a Gaussian mixture model (GMM) with two components; the last method is a simple threshold where a sample is classified whether its maximum is above or below bin 50. The resulting clustering is shown in the bottom of Fig. 8.

All three methods create the same two classes: one where most samples are above bin 50 and another where most samples are below bin 50. While the exact value of the simple threshold might seem arbitrary, it illustrates what PCA and the GMM are doing: they split a Gaussian at its mean. More generally, if we draw samples from a single distribution and ask a clustering algorithm to cluster them into two classes, these two classes tend to contain samples from the upper and lower end of that distribution, respectively.

This way of splitting a distribution is similar to what happens in the left column of Fig. 7 and also happens for other clustering algorithms.^17,20,44–46^ As alluded to with the GMM, splitting a Gaussian is not unique for unsupervised machine learning. It can also happen if we try to fit two Gaussians to a single, larger Gaussian.

We emphasize that the high- and low-conducting clusters exist and potentially corresponds to interesting physical phenomena. But, as shown by the change of distance metric, such a clustering result is one out of many. It should not necessarily constitute as evidence of any initial hypotheses, but rather, function as a starting point for further testing.

For example, as recently investigated through the use of molecular dynamics simulations,^61^ the broadness of 1D-histograms might be due to the stochastic nature of junction rupture. As each trace ruptures at a different displacement length, the main molecular peak will occur at slightly different conductance values. Such a situation is similar to what we have illustrated in Fig. 8.

We believe the biggest strength of clustering algorithms is their ability to help us generate interesting hypotheses; not to confirm them. We should perform additional, supplementary experiments to make sure that a given clustering result is robust.^62^

Even though we have shown some problematic aspects of clustering algorithms, we think their application in single-molecule transport studies is interesting and opens an exciting avenue to explore our data. They elegantly circumvent our need for labelled data, but we have to be careful not to introduce new problems or obscure current ones. As such, they should be seen as a way to explore our data and a way to generate hypotheses that can be investigated through future experiments rather than the clusters providing physical insight in and of themselves.

Analogous to the problem of variable clustering results are Rashomon sets.^63^ The term comes from a 1950 Kurosawa movie named “Rashomon” where four individuals describe the same episode in contradictory ways. In ML, Rashomon sets refer to different models having roughly the same performance. The “individuals” refer to the parameters of each model describing the data. These parameters can be wildly different even for models from the same class of models. Therefore, regardless of whether our model is supervised or unsupervised, we need to supplement our data analysis with experimental hypothesis testing.

### 3.5 Removing uninformative features (feature selection)

As explained in Section 2.1 “Feature extraction”, it can be desirable to construct a minimal set of relevant features that contain predictive information about our classes. A minimal feature set will help with the performance, training time, and sometimes also interpretability of the model. Knowing *a priori* which features are relevant can be difficult and, therefore, it is common to start with a large feature set that is subsequently pruned. In the following, we will show how such filtering is done, how it might help the performance of our models and lastly, where care is required.

**Fig. 9:**
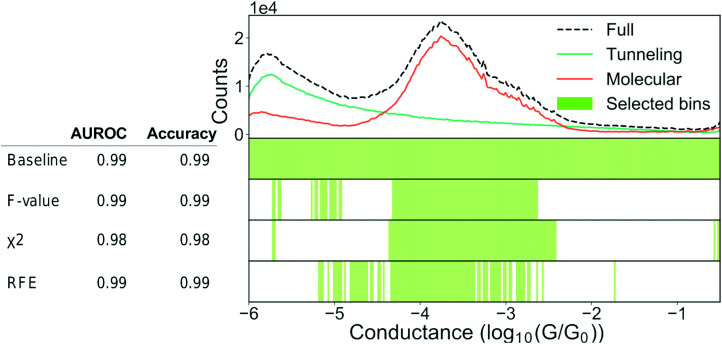
Feature selection can help our understanding. Top: 1D-histogram of the 4K-BPY data set where each trace was labelled manually. The green line is tunnelling traces, the orange line is molecular traces and the black, dashed line is the combined 1D-histogram of both tunnelling and molecular traces. Beneath the 1D-histogram are shown four barcode plots. “Baseline” is the full set of features; “ANOVA *F*-value” is the remaining features after filtering according to the ANOVA *F*-value between each feature and the target label; “*χ*^2^” is the remaining features after filtering according to the *χ*^2^ – value between each feature and the target label; “RFE” is recursive feature elimination where the classifier is recursively trained with a smaller and smaller subset of the original set of features. Each iteration removes the *k* lowest ranked features (in this example *k* = 2). To the left of the barcode plots is shown a table of the AUROC and accuracy for each set of features. For all three examples, we used a random forest classifier with default settings. The original set of features was 256 bins thinned to 96 bins. Both the classifier and the filtering functions can be found in the Python package scikit-learn.^64^

In Fig. 9, we show an example of how feature filtering might impact the performance of our models and the analysis of our data. We use the unbalanced 4K-BPY data set that we introduced in Section 3.3 “Building trust in our models”. We have used the scikit-learn implementation of a random forest (RF) model with default settings. A RF-model is a non-linear classifier and, as such, it can be difficult to understand which features the model relies on. As input, we have converted each trace into a 1D-histogram with 256 bins in the range −6*G*_0_ to −0.5*G*_0_.

At the top of Fig. 9, we show a 1D-histogram of the full data set (dashed, black line), the true molecular traces (orange line) and the true tunnelling traces (green line). Underneath the 1D-histogram, we have plotted four barcode plots. The green shaded area illustrates which bins have been selected by a particular feature selection-method. The baseline model achieves 0.99 AUROC and 0.99 accuracy score with a train/test split of 70/30 using the full feature set of 256 bins.

We test three different feature filtering-methods: ANOVA *F*-value, *χ*^2^ and recursive feature elimination (RFE). All three are implemented in scikit-learn.^64^ Another common method of feature selection is to impose *L*_1_ constraints on the loss function of a model though we will not use this method for practical reasons. It can be incorporated in most models though it can be cumbersome due to the changes to the loss function.

We reduce the number of features from 256 to 96. Despite this reduction, there is no significant drop in neither AUROC nor accuracy for any of the three methods. As there is no considerable drop in performance, the filtered features cannot have contributed any discriminatory information that is not already present in the retained features.

All three methods pick out features that are centred around the main peak at approximately −3.8. This makes sense, as we see from the 1D-histograms of the tunnelling and molecular traces that this is the region where the traces, on average, differ the most from each other. The feature sets are distinct for each of the three filtering methods and this might be due to cross-correlation between the features or simply because different feature filtering methods emphasise different parts of a given feature set. This distinctness means that feature filtering should be seen as a guiding principle as to which features that can represent what is important, and not as a method of obtaining the ground-truth feature set. It should also be noted that the retained features are merely correlated with the class label in a predictive manner and do not imply causality.

It is a common mistake when performing feature filtering to filter the features before the data has been split into a training and test set. If the split does not happen before we filter our features, we risk leaking information from the test set into the training set. Such a leak can skew the performance of our model away from its true performance.

**Fig. 10:**
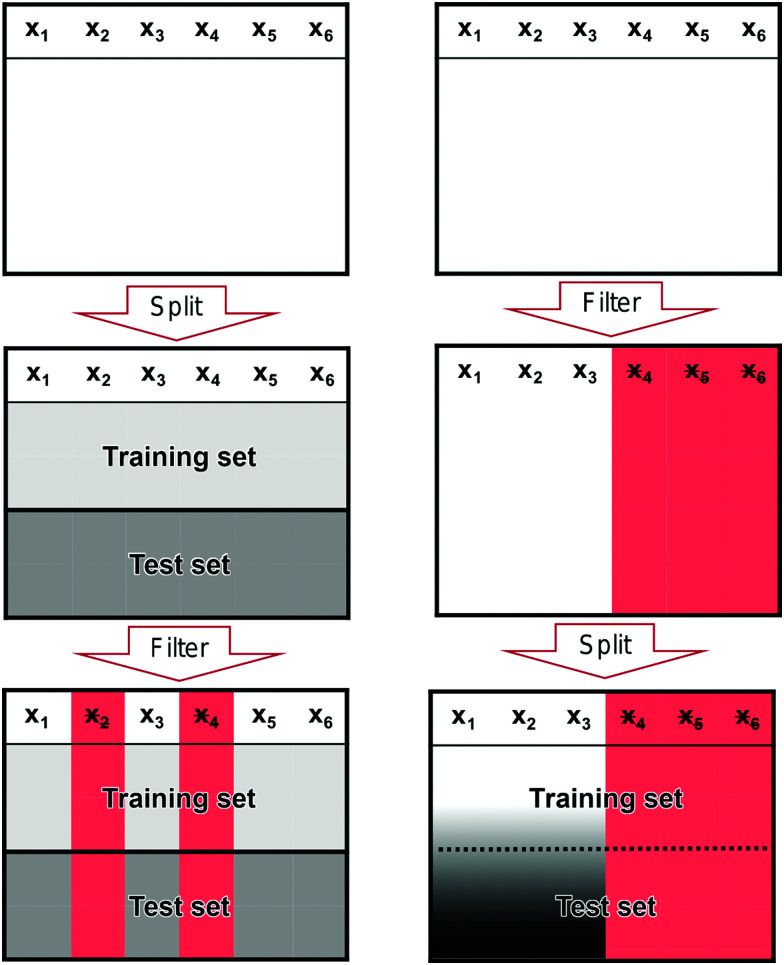
Illustration of the correct and incorrect way to do feature selection. (left) The correct way to perform feature filtering where the data set is split into a training and test set before filtering is performed. (right) The incorrect way to perform feature filtering where filtering is performed on the full data set before it is split into training and test set. The red columns mark features that have been removed and the black-white gradient illustrates that information from the test set has leaked into the training set.

In Fig. 10, we show the correct (left column) and incorrect (right column) way to perform feature filtering. In the left column, we start with the full data set at the top where *x*_1_, *x*_2_,…,*x*_*n*_ denote features. In single-molecule studies, these features could correspond to bins of a 1D- or 2D-histogram. We split the full data set into two sets: a training set and a test set. This split is illustrated by the division of the middlebox in the left column. In the last step, we filter features based only on the training set and then remove the same features from the test set as illustrated by the red columns in the last box.

In the right column, we show the incorrect way of filtering features. We start with the full data set, but, contrary to the correct case, we start by filtering features based on the full data set (red columns in the middlebox) before we split it into a training and a test set. Only after performing the filtering, we split the data set. Now, information in the test set has leaked into the training set as illustrated by the black-white gradient in the last box. This is a methodological flaw that has the potential to severely alter the perceived performance of a model without any warning signs.

In the ESI,† we show an example of how such information leakage might happen with a concrete code example.

As an example of how it might skew our results, we have created a synthetic data set of 12k samples that are all drawn from a single, multivariate Gaussian distribution. Each sample has a dimension of 32 × 32 = 1024 to mimic the number of features we would have if we convert a molecular trace to a 2D-histogram with 25 × 32 bins. Each sample is arbitrarily assigned a label of 1 or 0. We repeat this experiment 500 times. It should be clear that the performance of any model on this data set should be no higher than random chance, *i.e.*, accuracy of 50%.

**Fig. 11:**
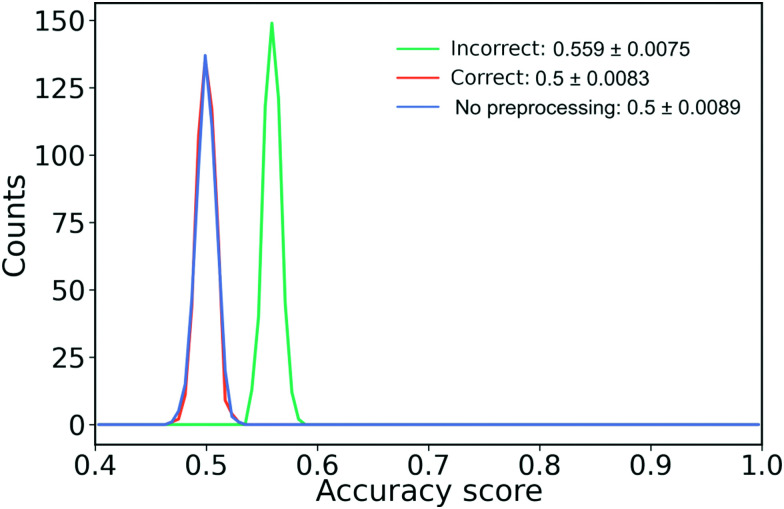
How information leakage from filtering features might lead to biased results. 12k samples drawn from a Gaussian distribution with a dimension of 32 × 32 are randomly assigned to class A or B leading to 6k samples in each class. “Wrong” (green line) perform feature filtering and scaling before splitting into test and training set, “Right” (orange line) splits the data, applies preprocessing on the training data and applies the learned preprocessing on the test data while “No preprocessing” (blue line) only splits the data into a training and test set. We perform 500 runs.

As we show in Fig. 11, when we perform feature filtering the correct way or do not perform any feature filtering, we have an accuracy of 50%. On the other hand, if we perform feature filtering the incorrect way, we artificially boost the performance by ∼6%. Note that we have about an order of magnitude more samples than features, yet our incorrect model still performs too optimistically. If we had fewer samples, the artificial increase in performance would have been bigger and *vice versa* for more samples. A related issue is doing feature filtering the correct way on a data set that has close to or more features than there are training samples.^65^

Here, we have described the issue of information leakage in the context of feature filtering though the issue is more general than that. It is a form of overfitting and, as mentioned in Section 3.2 “Overfitting supervised models”, it can also happen if we do not set aside part of the data before we start looking at it. Insidiously, information leakage can happen through the practitioner if they choose to look at the full data set before setting aside some of it as a holdout set and splitting the rest in a train and test set. In this way, we, the humans, might focus on spurious correlations that we impress upon our machines.

### 3.6 Sharing – of models and data

Being more forthcoming in sharing our work is another approach to alleviate the problems we have outlined and to help us grow as a community. Sharing of scripts for preprocessing and analysis,^66^ and of experimental data. Some groups are already sharing their code and data,^23,46,47^ but this needs to be more common. As explained in Section 3.1 “Defining bias”, there is a concern that human input skew our conclusions. While this is a fundamental problem in science that is not solved with ML techniques, part of the solution is to share our data.^67^ Such an open-source approach aligns with a recently proposed set of guidelines for ML in chemistry.^68^

This desire to share data has led to the principles for FAIR data that is **F**indable, **A**ccessible, **I**nteroperable and **R**eusable.^69^ FAIR aims to maximise the potential from data sets and maximise research impact. On a practical level, some studies indicate that open-source leads to an increase in citations.^70^ It also makes it easier for reviewers and readers to reproduce and validate a reported approach. Such reproducibility of shared code is important if we wish to avoid the replication crisis that artificial intelligence research is struggling with.^71^

In the ML community there seems to be a fundamental belief that all code should be open-source. This belief has led to services such as https://paperswithcode.com, free hosting of source code repositories like https://Github.com and extensive use of https://arxiv.org. This deep-rooted belief even sparked controversy surrounding the launch of Nature Machine Intelligence, a journal that was initially closed-source but is now optionally open access.^72^ Sharing also opens up new types of papers and is one of the main reasons for the creation of Open Reaction Database.^73^ For instance, studies on how different ML methods compare against each other, or more comprehensive studies on particular molecules.

We acknowledge that there are several challenges with sharing – both technical and personal. For example, the challenge of storing experimental data is significant. The data sets in single-molecule transport are often large, unstructured and there is no standardised way to provide metadata.

The availability of high-quality, labeled datasets has been a tremendous boon for the development of performant ML models. Such high-quality datasets would also benefit single-molecule transport studies, but even unstructured datasets are important to share with any published paper. Without access to the data that a paper is based on, it is impossible to independently verify the conclusions of that paper. For example, it can be valuable to test how robust the conclusions of a paper are to the specific choices made during data preparation.

We suggest that work be initiated on developing common protocols for sharing data. Increasingly, institutions have begun to offer hosting services, but there are also independent solutions such as https://academictorrents.com. Thinking about where to archive our work also forces us to find a long-term storage solution. Services such as Github have *de facto* solved the technical challenge of storing source code yet other challenges persist.

On a personal level, sharing source code can be very intimate. It is our creation and putting it out for everyone to see and to critique can be intimidating. It might also mean that we lose some ‘secret’ knowledge that gained us an edge. While we cannot offer any solutions to these softer problems, we feel that the gains of open-source outweigh the challenges.

There is also the problem of when to release data as we might feel that there are more papers to write about a given data set. Related to this problem is the concern of getting scooped. We acknowledge that this is a genuine reason for holding on to a data set. A solution could be that only part of the data set is released or that the group commit themselves to release the data at a later point.

Our attitude towards sharing can be summarised succinctly: more is better! As we have outlined, there are legitimate circumstances that prevent us from sharing the full extent of our work, but even a little goes a long way. The more we openly make available for each other, the quicker we can advance our field. Science is a collaborative endeavor and we should take full advantage of the communicative facilities that are available to us.

## 4 Where do we go from here?

In our opinion, the use of unsupervised learning should be seen as a way to generate hypotheses and not as a way to validate them. Given the nature of single molecule transport data, it is difficult to know *a priori* what each trace constitutes and even how many subpopulations there should be.

We feel that more work should explore how much and what kind of information we can extract from traces. Both in terms of new features, but also where in the trace we are looking. New representations of the data would benefit ML algorithms and researchers alike. Several groups have already begun work on this and we think this is an important direction to explore.^15,74–76^

A richer representation of the data would also enable more extensive use of traditional statistics. Perhaps an approach could be envisioned where newer machine learning techniques quickly filter out traces that has contaminants. More traditional statistics could then be used to summarise characteristics about the data or explore potential correlations.

One thing we have not touched on is the hard classification often employed when dealing with classes in single-molecule transport studies (also in this paper). Perhaps a softer classification could prove beneficial where a trace does not necessarily belong to a single class, but might exhibit characteristics of multiple classes. For example, some traces might be 80% molecular traces as they break later than traces that are only 20% molecular.

While we have not touched much on their use, tools for dimensionality reduction can facilitate a better understanding of a high-dimensional dataset. Many methods exist, but some popular ones include PCA, t-distributed stochastic neighbor embedding (t-SNE), or uniform manifold approximation and projection (UMAP). The use of t-SNE (and its variants such as UMAP) have been popular in recent years and some authors have started to investigate exactly when these methods break down.^77,78^

## 5 Conclusion

In this Tutorial Review, we have explored the exciting use and some of the challenges of ML in the field of single-molecule transport experiments. There is a need and a desire for more advanced data analysis techniques if we wish to gain further insight into our data. Advanced ML techniques potentially offer a solution, but we have to be careful. The powerful nature of ML comes with numerous pitfalls that require considerate caution and elaborate testing to avoid spurious claims. The problems we have highlighted and the opinions we have stated are mirrored by others.^68^

Alongside this manuscript, we have made many of the scripts and data available that we used to make our analyses and our figures. We encourage readers to explore both the data and the scripts – possibly even using the scripts to explore their datasets! They can be accessed at https://github.com/chem-william/TOM_paper. The data sets used in this paper can be accessed from https://erda.ku.dk/archives/23e862ff4a66f896a7ef635cbec16e0b/published-archive.html.

This manuscript, and the published scripts, has used Python as the language of choice. While Python offers a mature and rich ecosystem for ML and advanced data analysis, it is not the only one. For example, MATLAB, R, and Julia are all well-suited for ML.

The field of ML is in equal measure exciting and fast-paced. We believe its potential union with the field of single-molecule transport is an intriguing approach. Perhaps if an algorithm, a machine, can learn the intricacies of a molecular trace, maybe we can too.

## Conflicts of interest

There are no conflicts to declare.

## Supplementary Material

CS-051-D1CS00884F-s001
