# Supplementary material for: Trusting our machines: validating machine learning models for single-molecule transport experiments
Source: Chem Soc Rev. 2022 Jun 10;51(16):6875–92. doi: 10.1039/d1cs00884f (PMC9377421; doi:10.1039/d1cs00884f)
Supplement: CS-051-D1CS00884F-s001 [file CS-051-D1CS00884F-s001.pdf]

Electronic Supplementary Material (ESI) for Chemical Society Reviews.
This journal is © The Royal Society of Chemistry 2022

Supporting Information: Trusting our Machines: Validating Machine Learning Models for Single-Molecule Transport Experiments

William Bro-Jørgensen,[†] Joseph M. Hamill,[†] Rasmus Bro,[*,‡] and Gemma C. Solomon[*,†]

[†]*Department of Chemistry and Nano-Science Center, University of Copenhagen, Universitetsparken 5, DK-2100, Copenhagen Ø, Denmark*

[‡]*Department of Food Science, University of Copenhagen, Rolighedsvej 26, 1958 Frederiksberg, Denmark.*

E-mail: rb@food.ku.dk; gsolomon@chem.ku.dk

Considerations for risk of overfit

Relevance of the data: If you select a small number of features that you know are both essential and sufficient for describing your problem, the risk of overfitting may be small. If you measured high-resolution data containing information on thousands of variables, but do not know which variables are relevant for the classification task at hand then that is a recipe for problems.

Sample to variable ratio: If you have measured one feature and measured that on several thousand samples, it will be difficult to overfit. In the opposite case, having many variables

and few samples, you may easily overfit your data. In single-molecule transport experiments, we are often fortunate to be in the first case, yet that need not always be the case.

Complexity of the model: It is reasonable that the more flexible the model, the greater the potential for overfitting. Straight-line fitting has less risk of overfitting than a fifty layer neural network as illustrated with polynomials in Figure 5 in the main manuscript.

Complexity of the model construction: It is common to perform data analysis by making hundreds of choices on parameters such as deciding which variables to include, testing different types of preprocessing, comparing different models etc. This, in itself, is by no means wrong, but there are statistical repercussions. The risk of an overfit model increases with the number of parameters that are tweaked. It is analogous to the "look-elsewhere" effect or multiple comparisons problem where an observation is apparently statistically significant due to chance simply because of searching a large parameter space.[S1]

Other feature sets for clustering

Examples of clustering using the features described by Liu et al.[S2] As is clear, the results are dependent on both the chosen feature set and the distance metric. Some aspects that were noted in the main manuscript are also apparent here. For Euclidean distance (left column of Figure S1), we see that there is a major and minor class. For cosine distance (right column of Figure S1), we still see many different clusters. The clusters found using cityblock distance (middle column of Figure S1) have one cluster with a large molecular peak while the other cluster has a bimodal distribution.

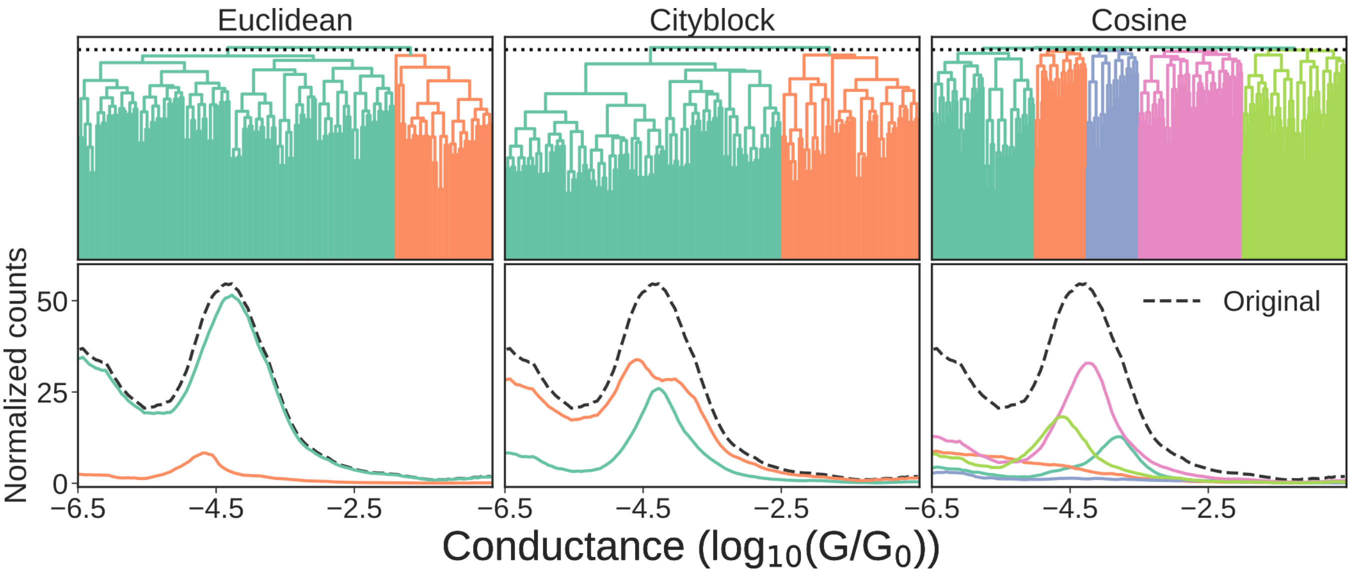

Figure S1: Clustering results on a one-molecule data set using complete-linkage and three different distance metrics: Euclidean, cityblock and cosine. Input features are 1D- and 2D-histograms concatenated to a single feature set. The top row shows the hierarchical clustering as a dendrogram and the bottom row shows the 1D-histograms for each cluster (colored lines) and the original dataset (black, dashed line). To condense the dendrogram, we omit some of the bottom nodes. This is merely done for the visualization and has no impact on the clustering result.

In Figure S2, we use 2D-histograms with 25×32 bins as input for the clustering algorithm. For there to be meaningful clusters, we have lowered the threshold for what constitutes a cluster compared with the threshold used in the main manuscript. That is, we use

$$0.87 \cdot \max Z. \tag{1}$$

Here, Z is the linkage matrix which contains cluster tree information. Exact implementation details can be found at the documentation for `scipy.cluster.hierarchy.linkage`.[S3]

In the main manuscript, we used

$$0.99 \cdot \max Z. \tag{2}$$

In Figure S2, we show the clustering result when using the threshold of the main manuscript.

The cosine distance metric still finds a multitude of clusters whereas there are only two clusters for Euclidean and cityblock distance. The majority of the traces end up in one cluster so we lower the threshold (according to Equation 1) to generate a richer cluster structure.

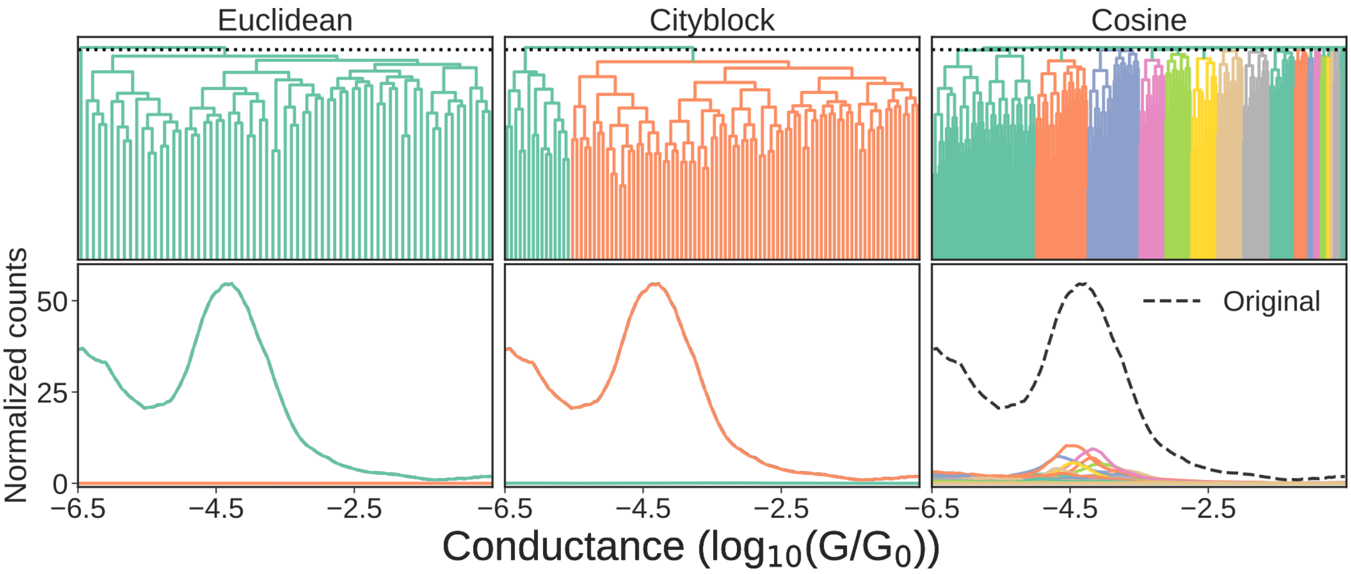

Figure S2: Clustering results on a one-molecule data set using complete-linkage and three different distance metrics: Euclidean, cityblock and cosine. Input features are 2D-histograms. Threshold is set according to Equation 2. The top row shows the hierarchical clustering as a dendrogram and the bottom row shows the 1D-histograms for each cluster (colored lines) and the original dataset (black, dashed line). To condense the dendrogram, we omit some of the bottom nodes. This is merely done for the visualization and has no impact on the clustering result.

When we lower the threshold (see Figure S3), we see that using the Euclidean distance matrix starts to split the main molecular peak into smaller peaks. According to the dendrogram, using the cityblock distance also generates more clusters though these are not apparent in the 1D-histogram.

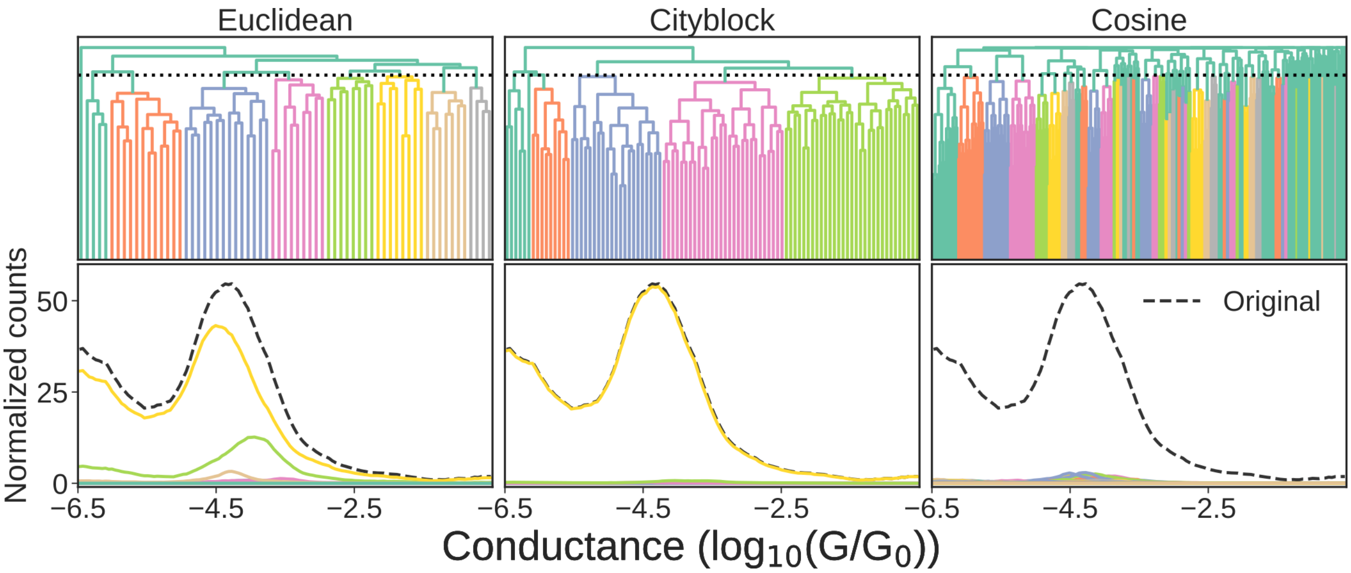

Figure S3: Clustering results on a one-molecule data set using complete-linkage and three different distance metrics: Euclidean, cityblock and cosine. Input features are 2D-histograms. Threshold is set according to Equation 1. The top row shows the hierarchical clustering as a dendrogram and the bottom row shows the 1D-histograms for each cluster (colored lines) and the original dataset (black, dashed line). To condense the dendrogram, we omit some of the bottom nodes. This is merely done for the visualization and has no impact on the clustering result.

Traces from 4K-BPY data set

In Figure S4, we show a handful of traces from the 4K-BPY dataset.[S4] The top row shows traces that have been labelled molecular and the bottom row shows traces labelled as tunneling.

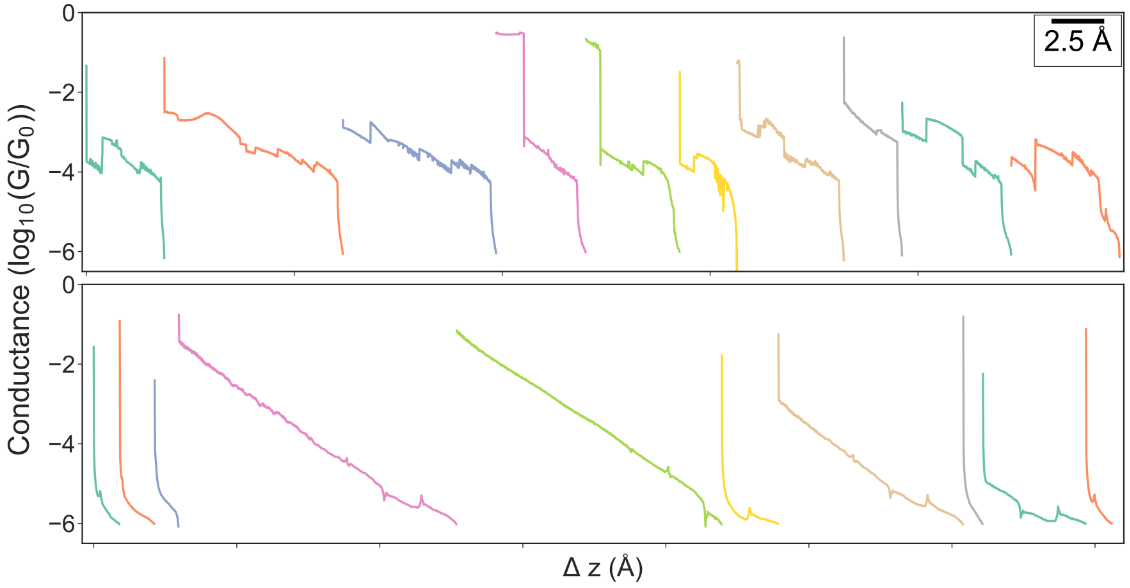

Figure S4: Examples of molecular and tunneling traces from the 4K-BPY dataset.[S4] The top row shows traces labelled as molecular and the bottom row shows examples labelled as tunneling.

Optimal model for 4K-BPY

In Figure S5, we illustrate how our choice of metrics might impact later analysis if we have an excellent model. Contrary to the main manuscript, we use a logistic regression model. As input for the model, we use 2D-histograms with 16×16 bins. For each trace, we have discarded any data point below $-6.5G_0$ and above $-1.5G_0$. After thresholding, we use the first 1250 data points.

From Figure S5(A), we can see that all models have an accuracy of 95% or above. Furthermore, the false positive rate is 7.6%, 1.5%, and 0.36% for the aggressive (grey), balanced (pink), and conservative (green) decision threshold, respectively. The true positive rate is 97%, 95%, and 91% for the aggressive, balanced, and conservative decision threshold, respectively

In Figure S5(B), we see that the choice between the three different decision thresholds

does not amount to a substantial difference. Only the 1D-histograms differ slightly from each other.

Ultimately, when all models perform well and similar to each other, it matters little for any later analysis. As can be seen from Figure S5(C), the length distribution from all three models are the same and they are all the same as the true distribution (black).

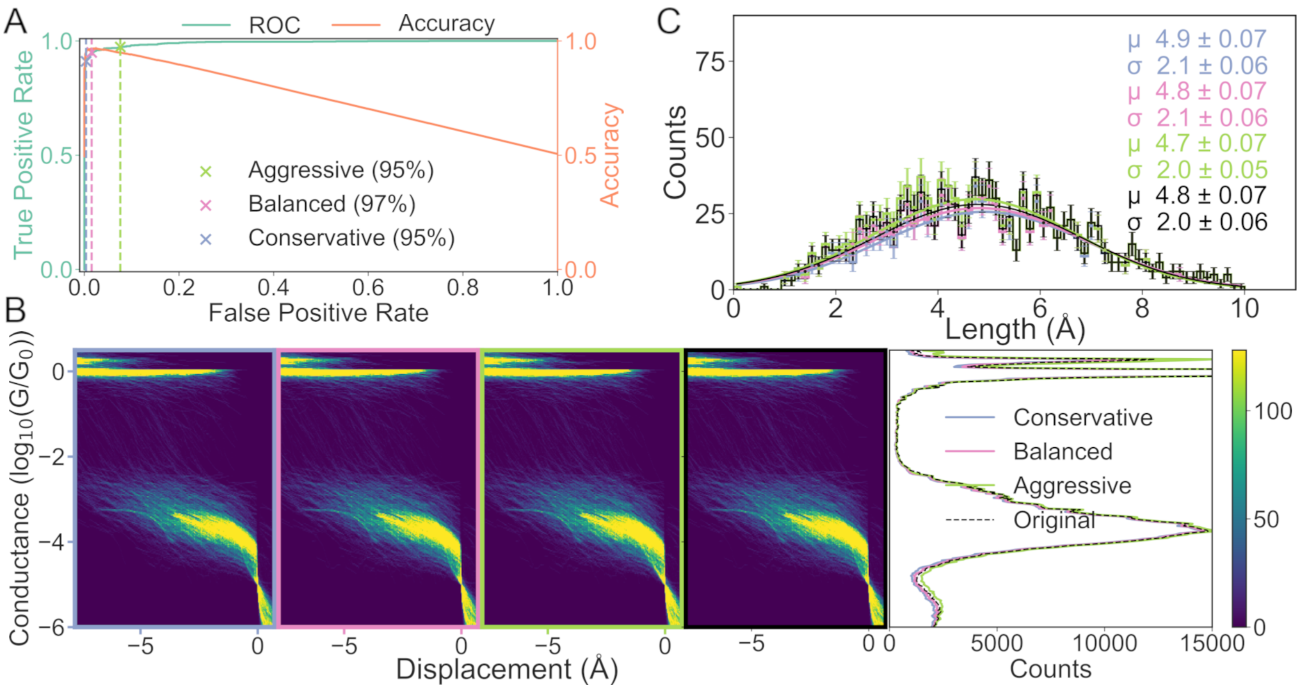

Figure S5: How choice of metrics impact later analysis. (A) False positive rate (FPR) vs. true positive rate (TPR) (green line) and FPR vs. accuracy (orange line). The three crosses and their corresponding dashed lines (grey, pink and light green) represent three different decision threshold levels: conservative, balanced and aggressive. The percentages in the legend lists the accuracy at each threshold. (B) Histograms of traces labelled "molecular". From left to right: Four 2D conductance vs. electrode separation histograms for the conservative, balanced, aggressive decision threshold and the true distribution, respectively. The red, dashed ellipsis highlights that a significant amount of tunneling traces has been misclassified as molecular. Final plot shows 1D conductance histograms at each decision threshold and for the true distribution. (C) Distribution of lengths of the molecular traces at each decision threshold at their respective colors and for the true distribution in black. The solid lines are fitted Gaussians with mean, sigma and error of each parameter given in the legend text.

Illustration of clustering data set

In Figure S6, we illustrate the clustering dataset used in the subsection titled "Predicting is not explaining" in the main manuscript. In the left column, we show individual traces and in the right column we show a 1D-histogram of the full dataset.

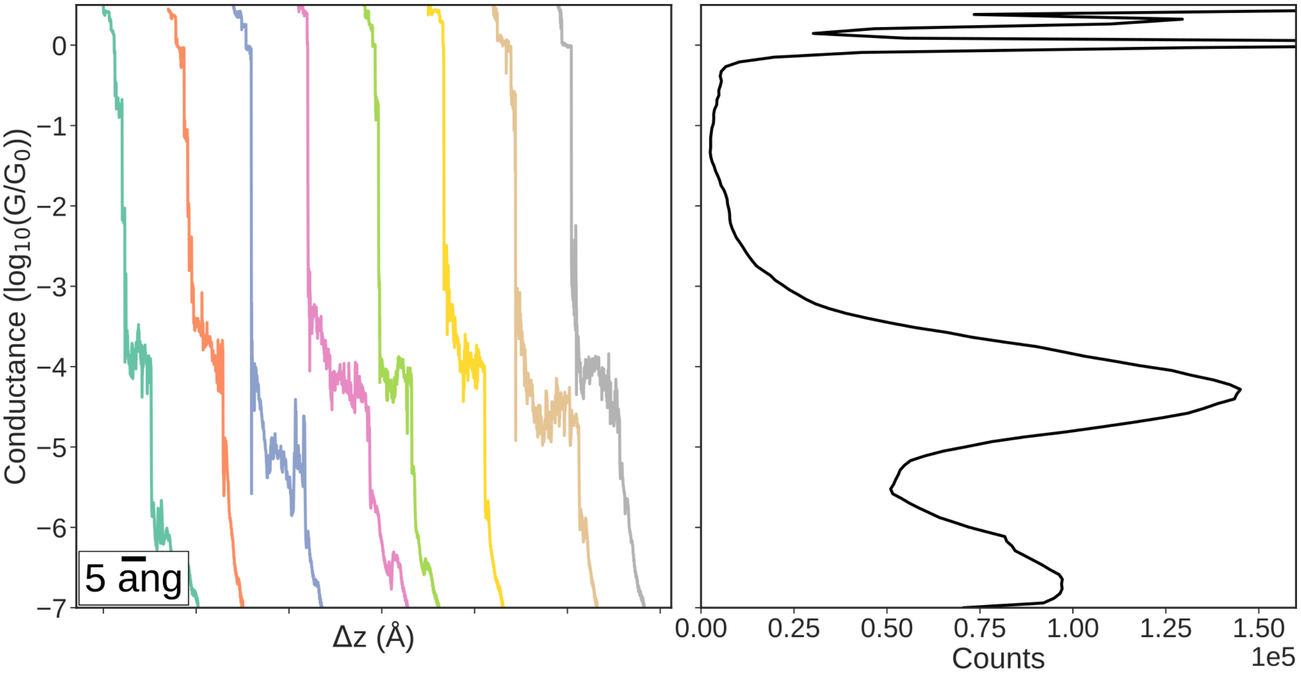

Figure S6: Examples of traces from the data set of the clustering dataset.[S5] The left column shows 8 traces from the full dataset. The right column shows a 1D-histogram of the full dataset.

Concrete feature filtering example

In Figure S7, we show a hypothetical example of the incorrect (left side) and correct way (right side) to perform feature filtering in Python. At the top of the figure, we import some of the functions that we will use in both examples. On the left side, the data is first filtered using `SelectKBest` from `scikit-learn`.[S6] The routine `SelectKBest` will select the k highest scoring features according to a given scoring function which, in this case, computes the analysis of variance (ANOVA) F-value for each sample. After the features have been filtered, the data set is split into a training and test set using the function `train_test_split()` that

is also provided by `scikit-learn`. The correct way to do feature filtering is shown on the right side. Here, the data is first split into a training and test set. After that, we use the `SelectKBest` routine on the training set to select the k highest scoring features. We can then remove the same set of features from the test set as we removed from the training set. In this way, no information has leaked from the test set into our training set as the decision about which features to remove are based solely on information available in the training set.

Note that, if the code is copied as-is, it will not run as the variables `X` and `y` need to be instantiated.

```
from sklearn.model_selection import train_test_split
from sklearn.feature_selection import SelectKBest
from sklearn.feature_selection import f_classif

#                         ...
#                         ...
#                         ...
# Load desired data set (X) and labels/responses (y)
```

```
### WRONG
kfilter_wrong = SelectKBest(score_func=f_classif)

# Filtering is performed on the WHOLE data set
X_transformed = kfilter_wrong.fit_transform(X, y)
X_train, X_test, y_train, y_test = train_test_split(
    X_transformed, y
)

# Further analysis...
```

```
### CORRECT
X_train, X_test, y_train, y_test = train_test_split(X, y)

kfilter_correct = SelectKBest(score_func=f_classif)

# Filtering is only performed on the training set
X_train = kfilter_correct.fit_transform(X_train, y_train)

# Without refitting, we use kfilter_correct
# that was fitted on the training data
X_test = kfilter_correct.transform(X_test)

# Further analysis...
```

Figure S7: The incorrect (left box) and correct way (right box) to perform feature filtering. On the left side, filtering is done before we split the data into a training and test set. On the right side, we first split the data into a training and test set, and then we perform feature filtering only on the training set. We then filter the features of the test set according to what we learned from the training set. Code is Python. Note that the code will not run if copy-pasted as the variables `X` and `y` have not been loaded.

Training and test errors on average

In Figure S8, we show the test error across 1024 experiments when we do the fitting as explained in Section 3.2 "(Over)fitting supervised models" in the main manuscript. As expected, the training error gets lower, when the polynomial order is increased. Despite this, only the third order polynomial has the lowest error as this matches the true data-generating model. While this fact holds on average, it is not necessarily true for every instance. Sometimes the higher order polynomials will have comparable or lower test error than the third polynomial.

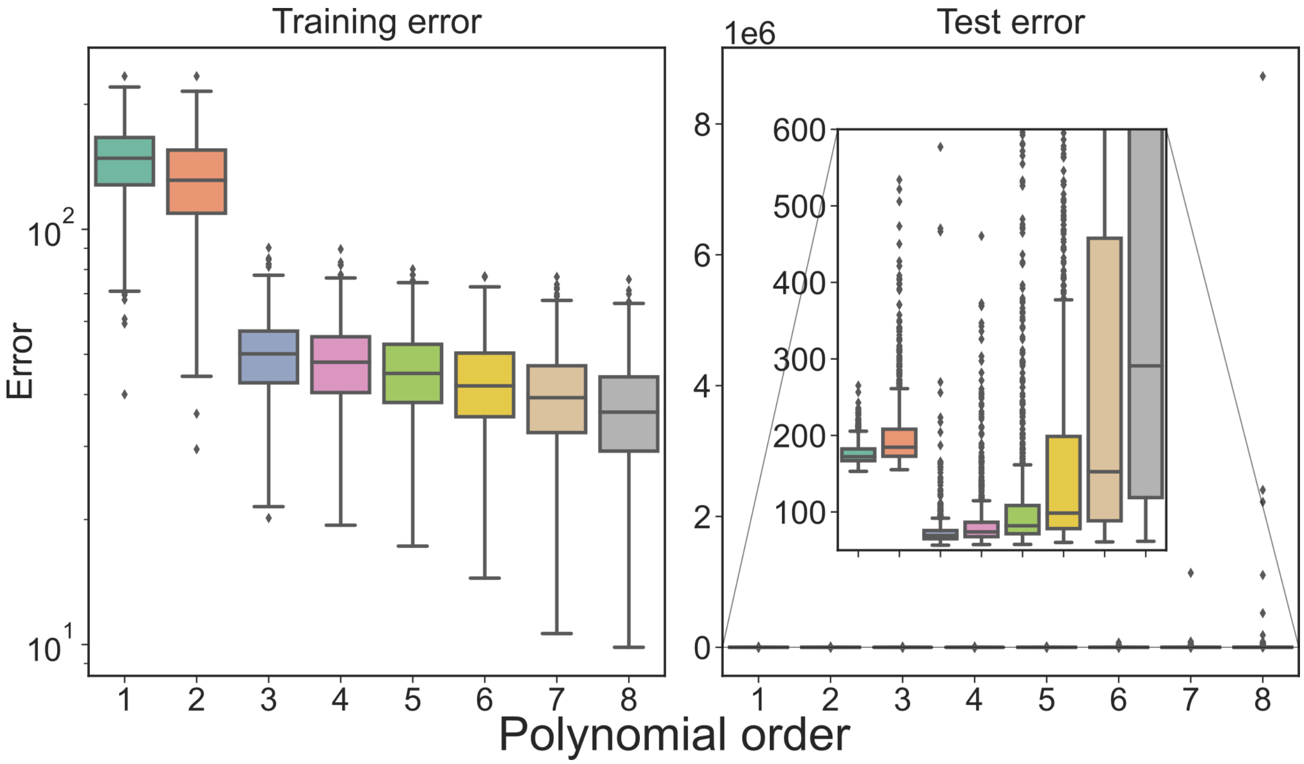

Figure S8: Example of repeating the fitting described in Section 3.2 "(Over)fitting supervised models" 1024 times. The whiskers extend 1.5 times the interquartile range. Any point outside this range is plotted as a diamond. Note the logarithmic scale. The inset shows a zoom-in on the test error as the higher order polynomials have a few samples with relatively enormous error.

References

(S1) Lyons, L. Open statistical issues in Particle Physics. *The Annals of Applied Statistics* **2008**, *2*, 887 – 915.

(S2) Liu, Y.; Ornago, L.; Carlotti, M.; Ai, Y.; El Abbassi, M.; Soni, S.; Asyuda, A.; Zharnikov, M.; Van Der Zant, H. S.; Chiechi, R. C. Intermolecular Effects on Tunneling through Acenes in Large-Area and Single-Molecule Junctions. *J. Phys. Chem. C* **2020**, *124*, 22776–22783.

(S3) Virtanen, P.; Gommers, R.; Oliphant, T. E.; Haberland, M.; Reddy, T.; Cournapeau, D.; Burovski, E.; Peterson, P.; Weckesser, W.; Bright, J.; van der Walt, S. J.; Brett, M.; Wilson, J.; Millman, K. J.; Mayorov, N.; Nelson, A. R. J.; Jones, E.; Kern, R.; Larson, E.; Carey, C. J.; Polat, İ.; Feng, Y.; Moore, E. W.; VanderPlas, J.; Laxalde, D.; Perktold, J.; Cimrman, R.; Henriksen, I.; Quintero, E. A.; Harris, C. R.; Archibald, A. M.; Ribeiro, A. H.; Pedregosa, F.; van Mulbregt, P.; SciPy 1.0 Contributors, SciPy 1.0: Fundamental Algorithms for Scientific Computing in Python. *Nat. Methods* **2020**, *17*, 261–272.

(S4) Magyarkuti, A.; Balogh, N.; Balogh, Z.; Venkataraman, L.; Halbritter, A. Unsupervised feature recognition in single-molecule break junction data. *Nanoscale* **2020**, *12*, 8355–8363.

(S5) Hamill, J. M.; Zhao, X. T.; Mészáros, G.; Bryce, M. R.; Arenz, M. Fast Data Sorting with Modified Principal Component Analysis to Distinguish Unique Single Molecular Break Junction Trajectories. *Phys. Rev. Lett.* **2018**, *120*, 016601.

(S6) Pedregosa, F.; Varoquaux, G.; Gramfort, A.; Michel, V.; Thirion, B.; Grisel, O.; Blondel, M.; Prettenhofer, P.; Weiss, R.; Dubourg, V.; Vanderplas, J.; Passos, A.; Cournapeau, D.; Brucher, M.; Perrot, M.; Duchesnay, E. Scikit-learn: Machine Learning in Python. *J. Mach. Learn. Res.* **2011**, *12*, 2825–2830.
